# Metagenomics reveals the temporal dynamics of the rumen resistome and microbiome in goat kids

**DOI:** 10.1186/s40168-023-01733-5

**Published:** 2024-01-22

**Authors:** Jianmin Chai, Yimin Zhuang, Kai Cui, Yanliang Bi, Naifeng Zhang

**Affiliations:** 1 Institute of Feed Research of Chinese Academy of Agricultural Sciences, Key Laboratory of Feed Biotechnology of the Ministry of Agriculture and Rural Affairs, Beijing, 100081 China; 2 Guangdong Provincial Key Laboratory of Animal Molecular Design and Precise Breeding, College of Life Science and Engineering, Foshan University, Foshan, 528225 China; 3 Department of Animal Science, Division of Agriculture, University of Arkansas, Fayetteville, AR 72701 USA

**Keywords:** Rumen resistome, Metagenomics, Microbiome, Ruminants, Ages, Temporal dynamics, Diet, Horizontal transfer

## Abstract

**Background:**

The gut microbiome of domestic animals carries antibiotic resistance genes (ARGs) which can be transmitted to the environment and humans, resulting in challenges of antibiotic resistance. Although it has been reported that the rumen microbiome of ruminants may be a reservoir of ARGs, the factors affecting the temporal dynamics of the rumen resistome are still unclear. Here, we collected rumen content samples of goats at 1, 7, 14, 28, 42, 56, 70, and 84 days of age, analyzed their microbiome and resistome profiles using metagenomics, and assessed the temporal dynamics of the rumen resistome in goats at the early stage of life under a conventional feeding system.

**Results:**

In our results, the rumen resistome of goat kids contained ARGs to 41 classes, and the richness of ARGs decreased with age. Four antibiotic compound types of ARGs, including drugs, biocides, metals, and multi-compounds, were found during milk feeding, while only drug types of ARGs were observed after supplementation with starter feed. The specific ARGs for each age and their temporal dynamics were characterized, and the network inference model revealed that the interactions among ARGs were related to age. A strong correlation between the profiles of rumen resistome and microbiome was found using Procrustes analysis. Ruminal *Escherichia coli* within Proteobacteria phylum was the main carrier of ARGs in goats consuming colostrum, while *Prevotella ruminicola* and *Fibrobacter succinogenes* associated with cellulose degradation were the carriers of ARGs after starter supplementation. Milk consumption was likely a source of rumen ARGs, and the changes in the rumen resistome with age were correlated with the microbiome modulation by starter supplementation.

**Conclusions:**

Our data revealed that the temporal dynamics of the rumen resistome are associated with the microbiome, and the reservoir of ARGs in the rumen during early life is likely related to age and diet. It may be a feasible strategy to reduce the rumen and its downstream dissemination of ARGs in ruminants through early-life dietary intervention.

Video Abstract

**Supplementary Information:**

The online version contains supplementary material available at 10.1186/s40168-023-01733-5.

## Background

In modern society, intensive livestock production systems provide high-quality milk and meat to meet the demand for sufficient and high-quality food, but it largely depends on the wide application of antibiotics that results in antibiotic resistance in animals and humans [1–3]. The rumen microbiome might act as a large reservoir of antibiotic resistance genes (ARGs) which have been defined as environmental contaminants since 2006 [4, 5]. Recently, several studies confirmed that ARGs (e.g., daptomycin, macrolide, betalactams, and aminoglycoside classes) widely exist in the rumen microbiome [4, 6–8, 8], and can be released into the feces and subsequently contaminates external ecosystems, such as water and soil, through runoff from manure, and be transmitted to humans by ruminant products or direct contact [9–13]. Thus, regarding the important position of the rumen microbiome in livestock production and its challenges as a reservoir for ARG dissemination, it is urgent to understand the association between rumen microbiome and resistome, thereby ensuring the sustainable development of animal husbandry and food safety [14].

Many variables may influence ARGs. For example, the use of antibiotics in disease treatment and feed additives can result in changes in ARGs [15]. The diet and environmental factors that can drive changes in microbiome dynamics or dysbiosis also have impacts on ARGs of the gut in both animals and humans [4, 7, 16]. Moreover, the horizontal transfer of ARGs is very important for ARG measurement since it allows for resistance to expand beyond specific clones. However, knowledge about the factors influencing horizontal gene transfer within the microbial community is limited [17]. Thus, understanding the sources, distributions, and residuals of ARGs within one community as a reservoir could help us integrate ARG dissemination among different systems. Auffret et al. concluded that diet affected the ARGs profile in the rumen microbial community in beef cattle as they found that chloramphenicol and microcin resistance genes were dominant in forage-fed cattle, but aminoglycoside and streptomycin resistances were enriched in concentrate-fed animals [4]. To explore the impact of dietary intervention on the development of antibiotic resistance is necessary since the dietary nutritional content is associated with gut resistome [18]. Compared to the fecal community, the rumen microbiome is distinct, as it experiences multifaceted and significant changes in the neonatal stage to acquire the capacity to digest carbohydrates [19–22]. Since the neonatal rumen microbiome is modulated by dietary factors [23, 24], it is necessary and urgent to investigate the characteristics of ARGs distribution and evolution in the rumen, and whether solid diet supplementation can affect the horizontal transfer of ARGs during the early life of ruminants, which is helpful to address the aggravation of bacterial resistance and dissemination.

To address this gap, we hypothesized that the early colonization of the rumen microbiome in newborn goat kids serves as an initial source of ARGs, and the rumen resistome is partly affected by microbial changes caused by diet during early life. In this study, metagenomics was performed to measure the temporal dynamics of the rumen resistome and microbiome in goat kids. Age-associated ARGs were identified by metagenomics, and the expression of these ARGs was measured by RT-qPCR and metatranscriptomics to validate the obtained results. Using network analysis, we further detected the associations among ARGs and their association with the rumen microbiota. Temporal changes in carbohydrate-active enzymes in the rumen microbiome correspondingly complement the association between dietary changes and resistome dynamics. The current research provides a comprehensive understanding of the rumen resistome in early life and reveals that diet during early life may modify ARGs in livestock.

## Results

### Ruminal microbiome changes with age and diet

**Fig. 1:**
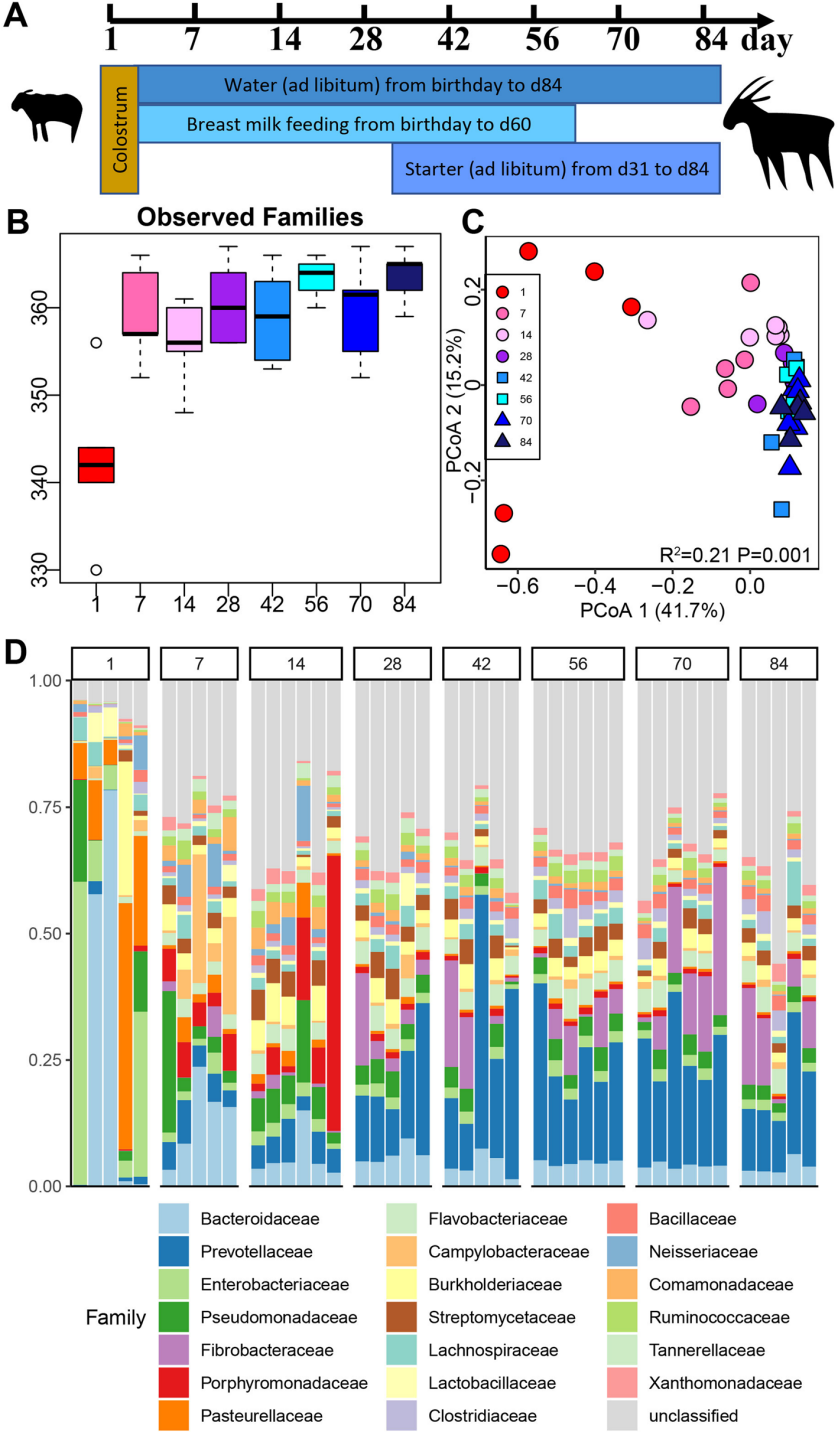
Rumen microbiome changes with ages in the early life of goats. **A** The ages, sampling day, and diet regime for goat kids in this study. **B** The number of observed families in rumen. The line inside the box denotes the median, and the boxes denote the interquartile (IQR) between the first and third quartiles (25th and 75th percentiles, respectively). The observed families on d1 were significantly lower than other ages (Wilcoxon rank-sum test, *p* < 0.05). **C** Principle Coordinate Analysis (PCoA) of Bray–Curtis distances between microbiota. The R2 and *P*-value of PERMONOVA to test the differences of beta diversity was labeled. The cluster of d1 samples was distinct compared with other ages, while d7 and d14 were clustered separately compared with the ages when goat kids accessed the starter diet (d42 to d84). **D** Bacterial abundances at the family level. Each bar represents a bacterial family and each column represents one sample

We sequenced the metagenome of 43 rumen-content samples of goat kids from 1, 7, 14, 28, 42, 56, 70, and 84 days old (Fig. 1A), which resulted in a total of 632 Gbps of sequencing data. The sequencing statistics are given in Table S1, and the taxonomic annotation rates are summarized (Figure S1). As microbiota is the major carrier for ARGs, the temporal dynamics of the rumen microbiome was first investigated to give fundamental knowledge. The rumen microbiota consisted of bacteria (94.6%), eukaryotes (3.2%), archaea (1.4%), and viruses (0.7%) (Table S2), which indicates that approximately 5% of the rumen is non-bacterial, reflecting a substantial level of cross-kingdom diversity. At the phylum level, Proteobacteria, Bacteroidetes, Firmicutes, and Actinobacteria were the four top taxa across all samples, accounting for 85% of the total bacterial community (Figure S2). Proteobacteria decreased in abundance from d1 to d28, while its level did not change from d42 to d84. Bacteroidetes remained at a similar abundance during the whole experiment. The abundance of Firmicutes varied at different ages, and it was higher on d28, d56, and d84. The abundance of Actinobacteria was low on d1 but remained high at other time points. Fibrobacteres increased in abundance over time and peaked after weaning (d70 and d84).

The observed families and Shannon Index on d1 were significantly lower than those at other ages (Fig. 1B, Figure S3). Sequencing data also displayed a temporal change in microbial structure based on principle coordination analysis (PCoA) (Bray–Curtis distance, PERMANOVA test: *R*^2^ = 0.21, *P* = 0.001) (Fig. 1C). The cluster of d1 samples was distinct compared with other ages, while d7 and d14 were clustered separately compared with the ages when goat kids accessed the starter diet (d42 to d84).

*Bacteroidaceae* and *Prevotellaceae*, two family members in the Bacteroidetes phylum, were major bacteria across all samples (Fig. 1D). The relative abundance of *Bacteroidaceae* was the highest on d1, followed by that on d7 and other ages with similar abundances. *Prevotellaceae* was among the multiple families with comparable abundance from d1 to d14, but exhibited a rising trend from d28 to d42, and remained a top family until the end of the trial. Another two important families belonging to the Proteobacteria phylum, *Enterobacteriaceae* and *Pasteurellaceae*, were enriched on d1 (21.2% and 16.7%), but their abundances were approximately 1% on other days. The *Fibrobacteraceae* (Fibrobacteres phylum) was approximately zero from d1 to d14 and significantly increased from d28 to d84. Moreover, some bacteria were enriched at certain ages. For example, the *Campylobacteraceae* (Proteobacteria phylum) was specifically abundant on d7, and *Porphyromonadaceae* (Bacteroidetes phylum) was enriched on d14.

The archaea in the rumen were also classified. The major phyla of archaea were Euryarchaeota, Crenarchaeota, and Thaumarchaeota (Figure S4A). The phylum Euryarchaeota accounted for 75% of total archaeal reads on d1 and over 80% on other days. Crenarchaeota and Thaumarchaeota were abundant on d1, while they were lower on other days. A total of 143 archaeal genera were observed, and the dominant genera across all samples included *Methanobrevibacter*, *Methanosarcina,* and *Thermococcus* (Figure S4B). The abundance of *Methanobrevibacter* increased from d1 to d14 and then decreased with increasing age.

### The temporal dynamics of the rumen resistome

**Fig. 2:**
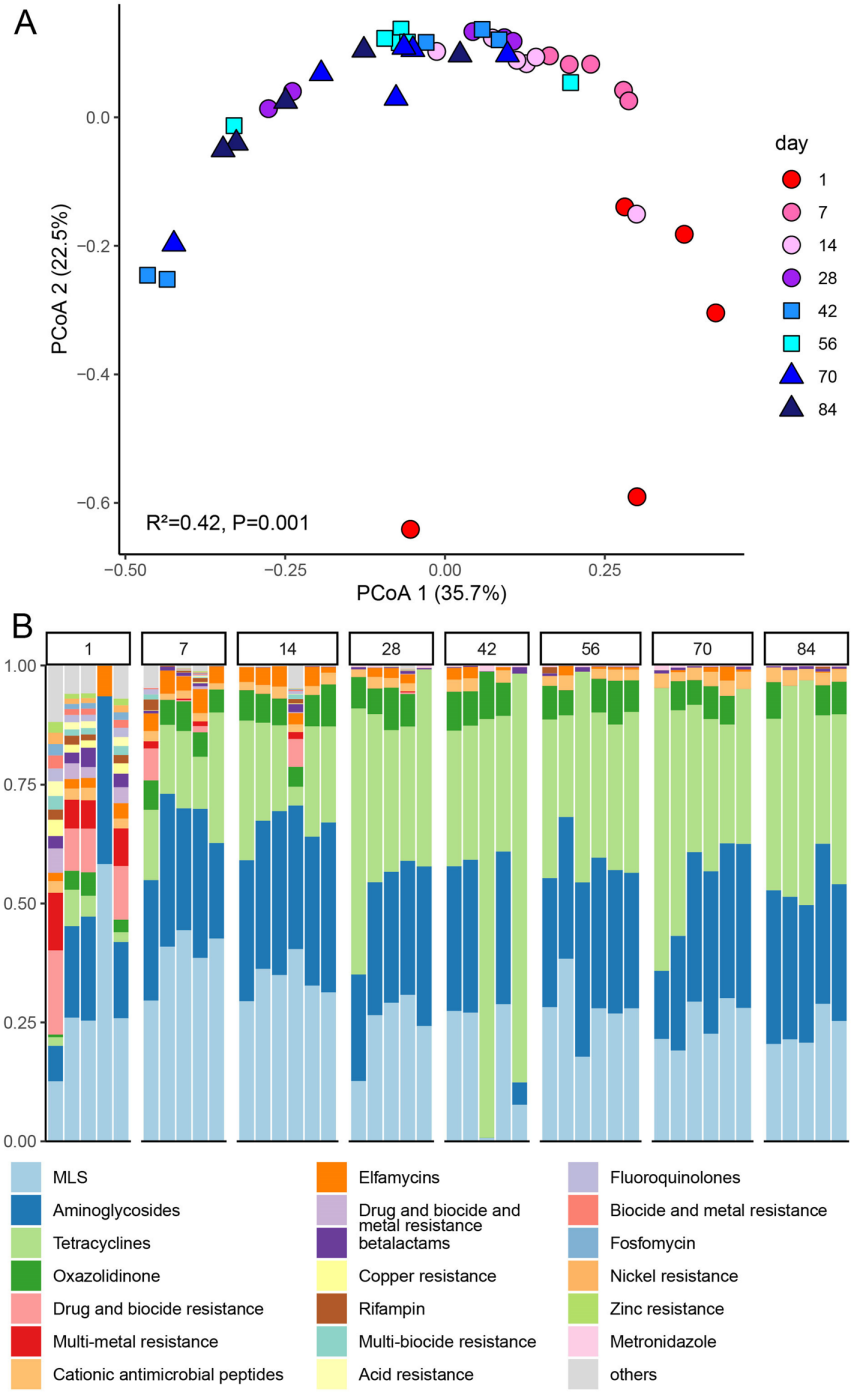
The rumen resistome structure and composition in goat kids. **A** Principle Coordinate Analysis (PCoA) of Bray–Curtis distances for the rumen resistome, showing changes in resistome structure over time as assessed by PERMANOVA test. Samples collected on d1 formed a separate cluster while later samples were more similar. **B** Relative abundance of antibiotic resistance genes (ARGs) at the class level of MEGARes 2.0. Each column represents a sample, and each bar represents an ARG class

To understand the temporal dynamics of the rumen resistome in goat kids during early life, metagenomic sequences were investigated for the presence of antibiotic resistance genes (ARGs). Our analysis with the AMRplusplus (v2) pipeline found that the 1031 detected ARGs belonged to 396 ARG groups across all samples, representing 41 antibiotic resistance classes. Four antibiotic compound types of ARGs, including Drugs, Biocides, Metals, and Multi-compound, were found from d1 to d28, while only Drug type ARGs remained from d42 to d84 in the rumen (Figure S5), indicating that the ARGs under Biocides, Metals and Multi-compound types possibly came from colostrum which is the primary nutrient source for goat kids but did not present in the rumen with age and diet. Similarly, the Shannon Index of ARGs was higher on d1 and kept a low value over time, while the richness of observed ARGs decreased significantly from d1 to d14 (*p* < 0.001) and remained at a similar richness from d42 to d84 (Figure S6). Moreover, the rumen resistome of goats significantly changed over ages, explaining the 35.7% variation based on the Bray–Curtis distance (PERMANOVA: *R*^2^ = 0.21, *P* = 0.001), with d1 samples forming a separate cluster while samples of later ages clustered together (Fig. 2A). Additionally, d7 and d14 samples were distinct from those of other ages when goat kids were supplemented with starter, which indicated that a change of the rumen resistome profile occurred at that time (Fig. 2A).

The ARG composition at the class level also changed with age (Fig. 2B). Corresponding to the diversity of ARGs, the number of abundant ARG classes was more on d1, while less dominant classes were observed from d7 to d84, with MLS (macrolides, lincosamides, and streptogramin A and B), Aminoglycosides and Tetracyclines accounting for approximately 89% of the total ARGs. Some ARG classes, including Drug and biocide resistance, and multi-metal resistance, were observed only on d1 after consuming colostrum. MLS increased from d1 to d7, and then decreased and maintained similar abundances from d28 until the end of this trial. The abundance of Elfamycins was approximately 3% from d1 to d14 and decreased from d42 to d84. The ARGs conferring resistance to Aminoglycosides, Tetracyclines, and Oxazolidinone increased on d7 and remained high until d84. The detected ARGs that were predicted to be Cationic antibiotic peptides remained at similar abundances throughout the experiment.

**Fig. 3:**
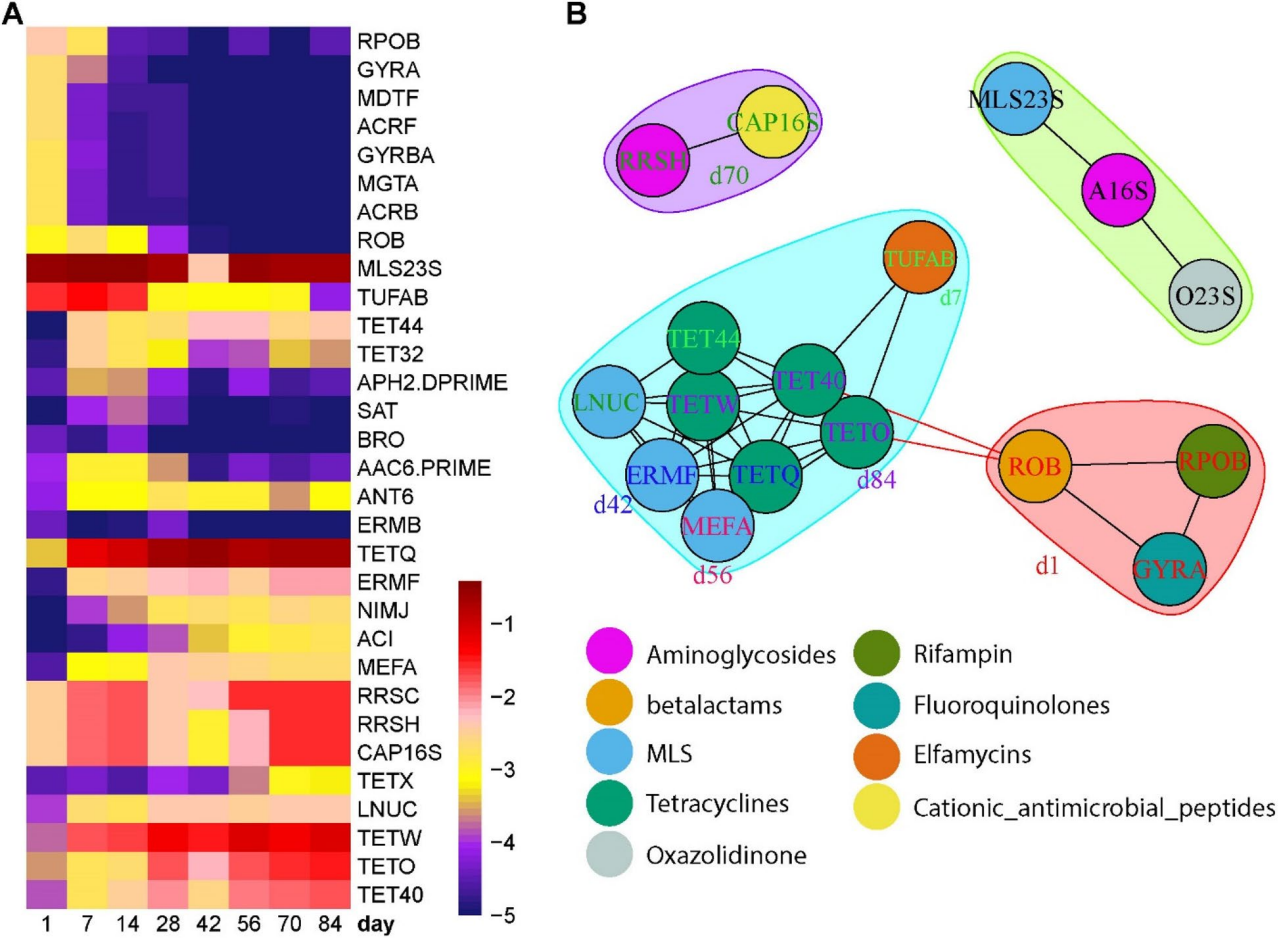
The temporal dynamics of antibiotic resistance genes (ARGs) and their interactions. **A** Heatmap depicted the age-associated ARGs identified by the LEfSe algorithm. The heat map shows the average relative abundances of ARGs on a log scale. The color of cells from purple to red corresponds to the relative abundance of ARGs from low to high. **B** A network analysis of the interactions among ARGs at different ages. The SparCC algorithm was employed for network analysis. The nodes (resistance genes, ARGs) were colored by antibiotics at the group level of MEGARes 2.0, and the font color of ARGs represents the age-associated signatures identified by the LEfSe

To determine the temporal variations in ARGs as the kids grew and the age-specific enrichment of ARGs, linear discriminant analysis (LDA) effect size (LEfSe) was performed (Fig. 3A). The ARGs, including four drug type ARGs (RPOB, GYRA, GYRBA, and ROB), three multi-compound ARGs (MDTF, ACRF, and ACRB), and one metal type ARG (MGTA), were all enriched on d1 and decreased in abundance from d7 to d84. The ARGs identified as the signatures for other ages belonged to the Drug types. On d7, the abundant ARGs were MLS23S, TUFAB, TET44, TET32, and APH2-DPRIME, while SAT and BRO were enriched on d14. Among them, TUFAB was abundant from d1 to d14 and remained non-abundant from d28 to d84. On d42, TETQ, ERMF, NIMJ, and ACI had the highest values, and their abundances were lower on d1 compared to other days. The abundance of MEFA reached a peak on d56 and decreased a little on subsequent days. The ARGs, including RRSC, RRSH, CAP16S, TETX, and LNUC, were enriched on d70, whereas TETW, TETO, and TET40 were abundant on d84. These signature ARGs for d70 and d84 increased abundance with age. Overall, the abundant ARGs on d1, such as RPOB, GYRA, GYRBA, MDTF, and ROB, decreased with age, but the dominant ARGs in the rumen were TETQ, TETX, TETW, TETO, and TET40 after supplementation starter. As a separate validation of the metagenomic dataset for ARGs, quantitative reverse transcription PCR (RT-qPCR) and metatranscriptomics confirmed that TET44, TETQ, TETW, TETO, and TET40, as major tetracycline resistance genes, increased with age (Figure S7). It indicates that the temporal changes of rumen ARGs in metagenomics were consistent with their expression.

To understand the temporal dynamics of ARG interactions in the rumen, the major ARGs were used to predict co-occurrence patterns. The network inference model revealed that the interactions among ARGs were strongly associated with age (Fig. 3B). ROB, RPOB, and GYRA identified as the d1-signature ARGs formed a cluster, representing the co-occurrences possible from colostrum effects. ROB was connected with TET40 and TETO which increased with age, which built a bridge to connect the dominant ARGs at different ages. The ARGs, which were higher during the period of starter supplementation and belonged to Tetracyclines and MLS, had strong correlations and formed the largest cluster. Moreover, a cluster of ARGs (RRSH and CAP16S) dominant on d70 was observed, and MLS23S with high abundances across all ages clustered with two other ARGs.

### Rumen microbial phylogeny associated with the resistome

**Fig. 4:**
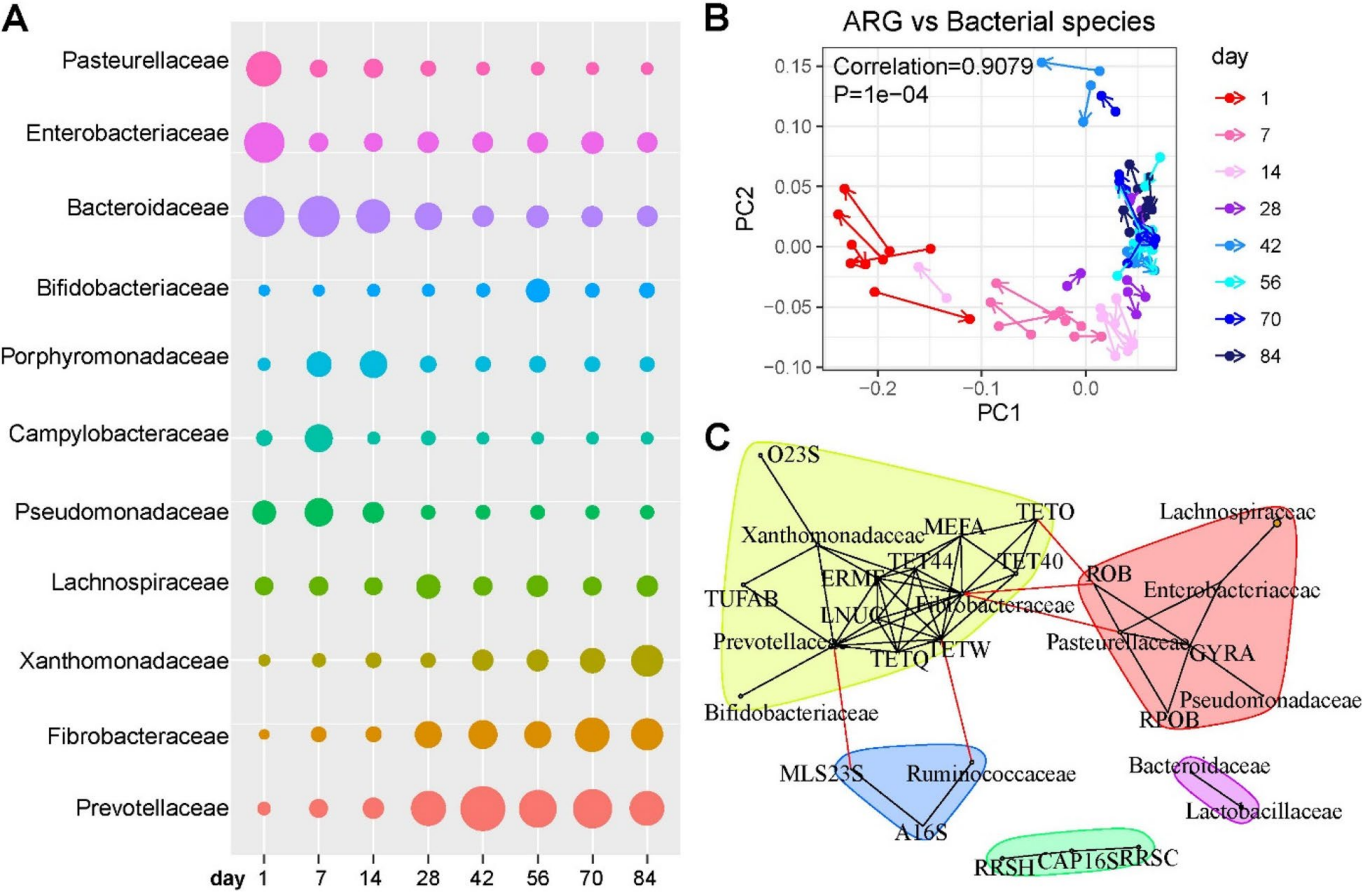
Rumen resistome associated with its bacterial community. **A** The most abundant host bacterial families of ARGs. Dots’ size represents the average relative abundance of bacteria at a certain age. **B** Procrustes analysis of the association between the composition of the resistome and that of bacterial community among different ages. The correlation coefficient, r, and *P*-value were generated by the ‘protest’ function, with *p* < 0.05 as the significant threshold. **C** A network analysis of the co-occurrence patterns between ARG and microbial taxa. The SparCC algorithm was used to calculate the relationships between bacterial taxa and ARGs. High abundances of families of Proteobacteria, such as *Enterobacteriaceae*, *Pasteurellaceae,* and *Pseudomonadaceae*, on d1, were strongly correlated with ARGs (ROB, GYRA, and RPOB) enriched. The abundance of bacterial families (*Xanthomonadaceae*, *Prevotellaceae,* and *Fibrobacteraceae*) increased with age and were associated with the ARGs as signatures during the starter supplementation period (d42 to d84)

By assigning taxonomy to ARG-containing metagenomic contigs, the microbial origin of observed ARGs in the rumen was identified. The bacterial host phyla of ARGs were mainly composed of Proteobacteria, Bacteroidetes, Firmicutes, Fibrobacteres, and Actinobacteria, and changed with age (Figure S8). Proteobacteria mainly carried ARGs on d1, and its abundance decreased and remained stable from d28 to d84. Bacteroidetes was another important carrier of ARGs on d1, and their abundance increased from d7 to d84. Fibrobacteres of ARGs was significantly increased from d42 to the end of the study. Proteobacteria, one of the most important ARG carriers, was associated with colostrum and starter supplementation. *Enterobacteriaceae* and *Pasteurellaceae* abundances were enriched on d1 (Fig. 4A). *Bacteroidaceae* was high on d1 and d7 and then decreased with age. However, *Xanthomonadaceae* was low from d1 to d28 and increased from d42 to d84. *Prevotellaceae* and *Fibrobacteraceae* increased significantly from d0 to d28 and remained in high abundance until d84.

A significant correlation between the composition of microbial communities and that of ARG profiles was confirmed by Procrustes analysis (correlation coefficient *r* = 0.9079 and *p* = 0.0001) (Fig. 4B), suggesting that communities with similar microbial compositions had similar resistome. Then, we investigated the co-occurrence patterns between ARGs and the major microbial families using the network analysis approach. High abundances of families of Proteobacteria on d1, such as *Enterobacteriaceae*, *Pasteurellaceae,* and *Pseudomonadaceae*, were strongly correlated with ARGs (e.g., ROB, GYRA, and RPOB) enriched on d1 (Fig. 4C). The bacterial families (e.g., *Xanthomonadaceae*, *Prevotellaceae,* and *Fibrobacteraceae*) increased with age and were associated with the ARGs as signatures during the starter supplementation period (d42 to d84). These taxa were speculated to be possible ARG hosts during the development of the rumen resistome in goats in early life. Moreover, *Prevotellaceae* and *Ruminococcaceae* formed a connection for MLS23S to other ARGs abundant during the starter feeding period, indicating ARG transmission among different ages.

**Fig. 5:**
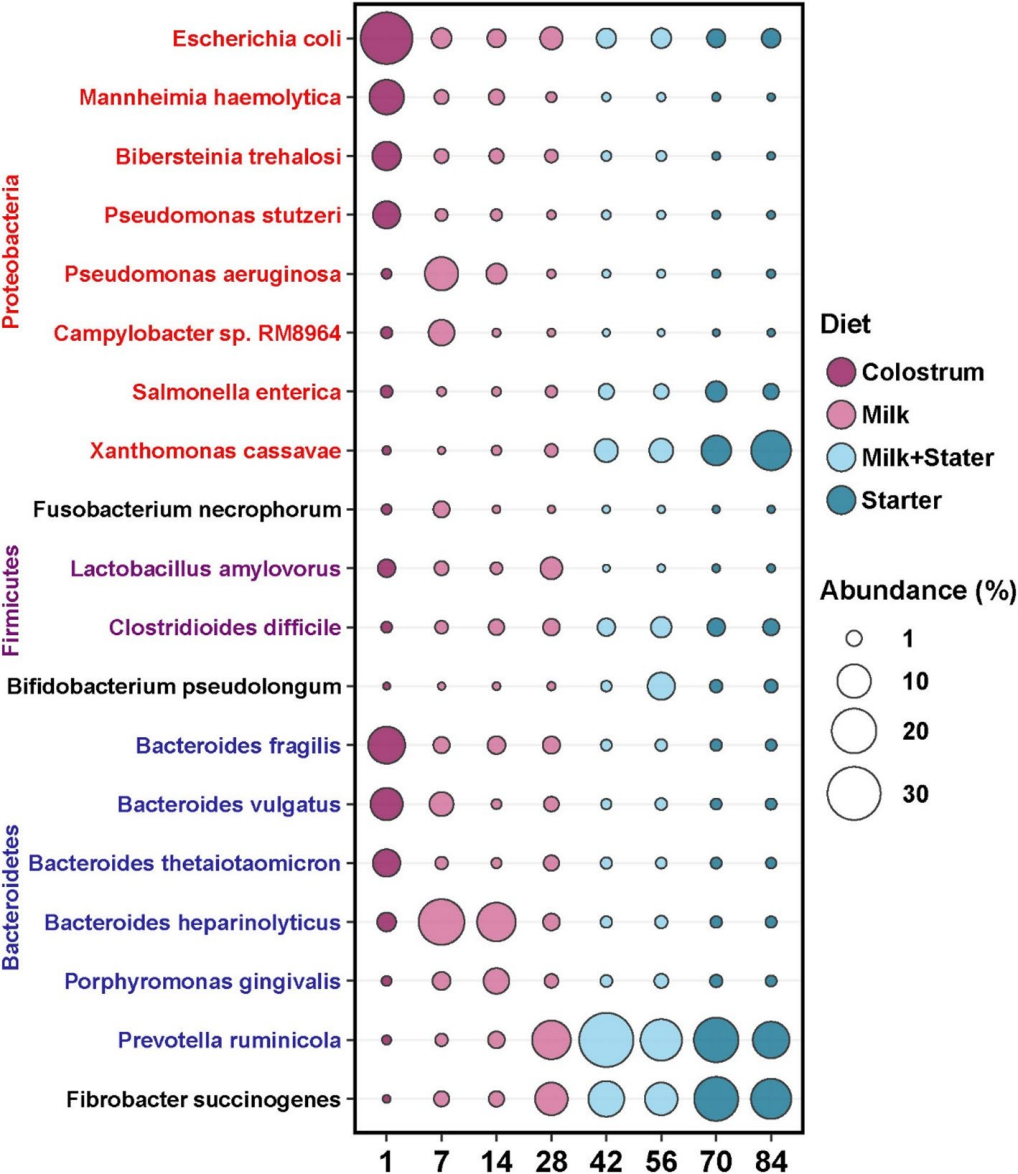
The temporal dynamics of majorhost bacterial species and phyla of antibiotic resistance genes (ARGs) in rumen resistome. The size of cycles represents the relative abundance of bacterial species at a certain age. To facilitate viewing, only those dominant species carrying ARGs were shown, and the diet regime was labeled with different colors for each microbial taxa. As observed, abundances and species of the bacteria carrying ARGs changed with age from d1 to d84

To better understand the bacteria carrying ARGs, the metagenomic contigs at the species level that were abundant at the families or phyla were characterized (Fig. 5). The main ARG contributors in Proteobacteria included *Escherichia coli*, *Mannheimia haemolytica*, *Bibersteinia trehalosi*, *Pseudomonas stutzeri*, *Pseudomonas aeruginosa*, and *Campylobacter sp. RM8964*, which mainly carried ARGs on d1 (colostrum stage) and d7 (milk stage), and *Salmonella enterica* and *Xanthomonas cassava*, which carried ARGs after d42 (period supplemented with starter). Several bacterial species under Bacteroidetes showed a similar pattern to Proteobacteria. For instance, *Bacteroides fragilis*, *Bacteroides vulgatus*, *Bacteroides thetaiotaomicron*, *Bacteroides heparinolyticus*, and *Porphyromonas gingivalis* were abundant from d1 to d14, while *Prevotella ruminicola* mainly carried ARGs from d28 to d84. Interestingly, *Fibrobacter succinogenes*, the only species in the Fibrobacteres phylum for the degradation of plant-based cellulose, increased significantly after supplementation of the starter (d42 to d84). Moreover, pathogens, such as *Fusobacterium necrophorum*, *Lactobacillus amylovorus,* and *Clostridioides difficile*, were characterized as ARG carriers, and their abundances were affected by age and dietary changes. Overall, *E. coli*, *M. haemolytica*, *B. trehalosi*, *P. stutzeri*, *B. fragilis*, *B. vulgatus,* and *B. thetaiotaomicron* were ARG hosts in goat rumen possibly due to consumption of colostrum. At the same time, *X. cassava*, *P. ruminicola,* and *F. succinogenes* carried ARGs when goat kids started to consume a high carbohydrate diet (starter), which indicated that colostrum served as the source for the initial rumen ARGs and bacterial carriers for ARGs were associated with age and dietary changes.

### Age and diet changed the functions of the microbiome and rumen resistome

Regarding the dietary transition from colostrum to milk to starter shaping the microbiome and the temporal dynamics of resistome in the rumen, the functions of the diet-related microbiome with age and its association with ARG structure were evaluated. After metagenomic assembly (statistics are presented in Table S3) and open reading frames (ORFs) prediction, we firstly annotated the functional and metabolic pathways of the rumen microbiome nonredundant gene catalog based on the Kyoto Encyclopedia of Genes and Genomes (KEGG) and eggNOG databases. From KEGG annotation results, the categories of ‘Metabolism’ and ‘Genetic Information Processing’ at level 1 were the most abundant across all ages (Figure S9). At the KEGG level 2 ortholog groups, many functions belonged to amino acid metabolism (18.23%), carbohydrate metabolism (13.32%), translation (11.66%), replication and repair (6.86%), membrane transport (6.58%), metabolism of cofactors and vitamins (5.72%), and nucleotide metabolism (5.23%) (Figure S10A). Moreover, eggNOG annotation showed that Posttranslational modification, protein turnover, chaperones (9.18%), and Carbohydrate transport and metabolism (6.03%) were more abundant (Figure S10B). Additionally, metatranscriptomics validated the main functions at KEGG level 2 obtained from metagenomics, such as amino acid metabolism, carbohydrate metabolism, and energy metabolism (Figure S10C).

**Fig. 6:**
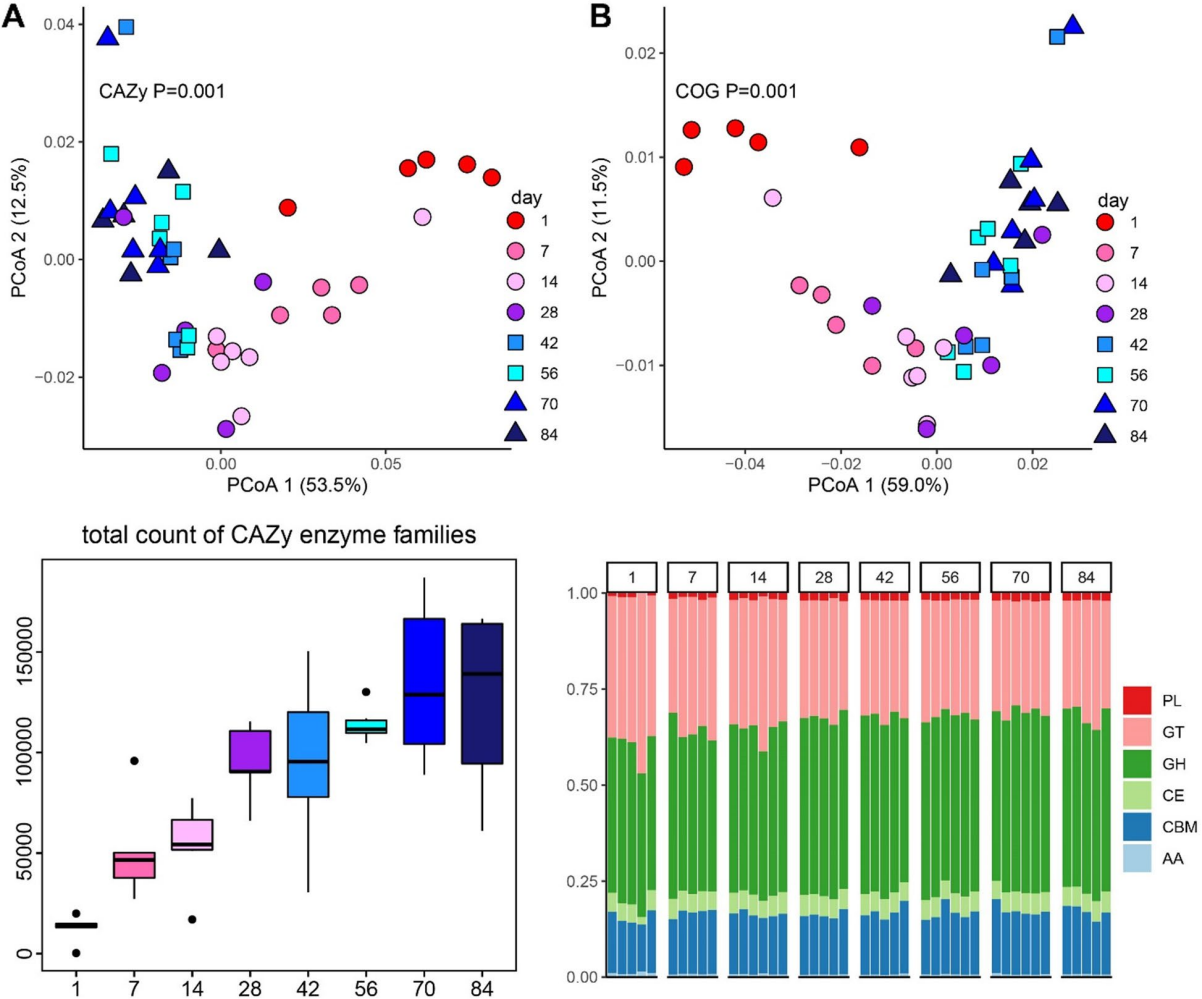
Changes in enzymatic activity over age. **A** Principle Coordinate Analysis (PCoA) of Bray–Curtis distance of CAZy content. **B** PCoA of Bray–Curtis distance of COG pathways. Each point in (**A**, **B**) represents a unique sample. The *P*-value of the PERMONOVA test was labeled. **C** Total count of CAZy enzyme families in goat kids from day 1 to day 84. **D** Stacked bar plot of the relative abundances of CAZy families per class of enzymes with age. Each column represent a sample. Carbohydrate-Active EnZymes database (CAZy); Clusters of Orthologous Groups of proteins database (COG); Glycoside Hydrolases (GH); GlycosylTransferases (GT); Polysaccharide Lyases (PL); Carbohydrate Esterases (CE); Carbohydrate-Binding Modules (CBMs); Auxiliary Activities (AA)

Then, we focused on the functions of carbohydrate-active enzymes and proteins. Rumen metagenomes analyzed for CAZy, COG, and KEGG showed a significant change in the functional configuration of the rumen microbiome with ages (Bray–Curtis; PERMANOVA *R*^2^ = 0.60, *R*^2^ = 0.59; *P* = 0.001 for both cases), with significant differences occurring on d1 as well as differences on d7 and d14 compared to other ages (Fig. 6A, B, Figure S11). A progressive increase in the number of total CAZy enzyme families was observed (Fig. 6C), and the count of each CAZy class increased with age (Figure S12). The predicted function of the rumen microbiome was composed of six classes of CAZy-annotated enzymes, of which over 76% characterized CAZy proteins were classified as glycoside hydrolase (GH) and glycosyltransferase (GT) (Fig. 6D). The relative abundance of GH increased from d1 to d28 and remained at this high level until d84, while GT had an opposite pattern compared with that of GH. The abundance of carbohydrate-binding modules (CBM) was approximately 15% among all ages. Genes encoding polysaccharide lyases (PL) were less abundant in d1 rumen samples but remained relatively abundant from d7 to the end of the trial. As an additional validation of metagenomics, metatranscriptomic data confirmed that the number of total CAZy enzyme families increased with age, and the major classes of CAZy-annotated enzymes were GH, GT, and CBM (Figure S13).

**Fig. 7:**
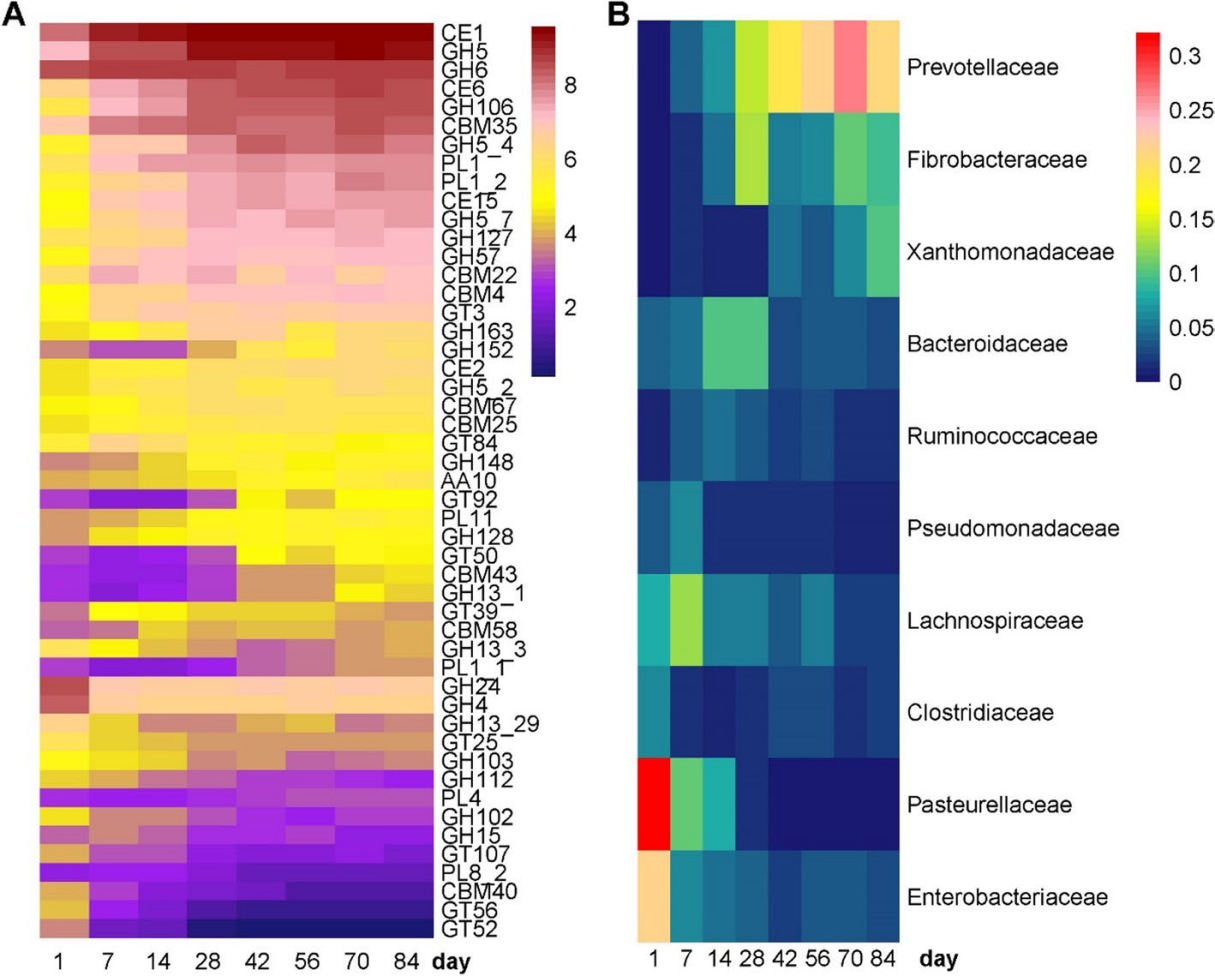
The abundances of age-associated CAZy families and their predicted bacterial families. **A** Heatmap depicting the age-associated CAZy enzymes. The color of cells from purple to red corresponds to the relative abundance of ARGs on a log scale from low to high. GH24 included lysozyme (EC 3.2.1.17) which is the major enzyme in colostrum. GH5 and GH6 contained cellulase (EC 3.2.1.4) and cellobiohydrolase (EC 3.2.1.91), GH5 included β-glucosidase (EC 3.2.1.21), and GH57 had α-amylase (EC 3.2.1.1). **B** The most abundant host bacterial families predicted to produce the CAZy are shown in the heatmap. The predicted bacteria, including *Pasteurellaceae*, *Enterobacteriaceae,* and *Clostridiaceae*, were abundant on d1, matching the abundances of the ARGs bacterial origins. The contributions of *Prevotellaceae*, *Fibrobacteraceae,* and *Xanthomonadaceae* to CAZy enzymes were high in later days, likely resulting from the increased intake of the solid diet

Differential abundance analysis was performed to identify CAZy families that changed with age in the rumen, most of which were GH and GT (Fig. 7A). GH24, GH4, GH107, GH15, GH102, GH112, GH13, GT52, GT56, and GT25 showed high abundances on d1. Among these enzymes, GH24 included lysozyme (EC 3.2.1.17) which is the major enzyme in the colostrum of goats. Other enzyme families including GH5, GH6, GH57, GH106, GT3, GT84, GT50, and GT25 increased with age and had higher abundances from d42 to d84. GH5 and GH6 contained cellulase (EC 3.2.1.4) and cellobiohydrolase (EC 3.2.1.91), GH5 included β-glucosidase (EC 3.2.1.21), and GH57 had α-amylase (EC 3.2.1.1), which indicated that CAZy families in the rumen were associated with the consumption of a starter diet containing high carbohydrate components. To validate the CAZy enzymes identified by metagenomics, the activities of α-amylase and carboxymethyl cellulase (CMC) in rumen were determined (Figure S14A, B). With age, the concentrations of α-amylase and CMC showed similar trends to the abundances of GH57 and GH6 in metagenomics (Figure S14C, D) and metatranscriptomics (Figure S14E, F), indicating the accuracy of metagenomic annotation of rumen enzymes. Moreover, metagenomic assembly was used to predict the bacterial origins of the observed CAZy families, and the most abundant microbiota at the family level changed with age as shown in Fig. 7B. The predicted bacteria, including *Pasteurellaceae*, *Enterobacteriaceae,* and *Clostridiaceae*, were abundant on d1, matching the abundances of the ARGs bacterial origins. *Lachnospiraceae* contributed to the CAZy enzymes on d7 and d1. *Bacteroidaceae* were abundant in d14 and d28. The contributions of *Prevotellaceae*, *Fibrobacteraceae*, and *Xanthomonadaceae* to CAZy enzymes were higher in later days, likely resulting from age and the increased intake of a solid diet. Moreover, these three bacterial families were the main ARG hosts from d42 to d84, reflecting that the function of the rumen microbiome is associated with the resistome.

The changes in CAZy enzymes and their predicted microbial hosts revealed that diet (both colostrum and starter) modulated the rumen microbiome harboring enzymes to digest nutrients (e.g., lactose and fiber). The absence of colostrum in the rumen and the presence of a new carbohydrate source from the starter decreased ARG species while age increased its abundance due to the changing rumen microbiome (Fig. 1D). The predicted microbial hosts of significantly changed CAZy enzymes corresponded with gradual changes observed in the rumen microbiome of ARGs as well as the network analysis at the bacterial family level (Fig. 4A and Figure S15). The relative abundances of *Prevotellaceae* and *Xanthomonadaceae* were negatively correlated with *Pasteurellaceae* and *Lachnospiraceae* (*r* =  − 0.52, *r* =  − 0.51, *p* < 0.05), respectively. Together, the data in this study support that both age and diet contribute to the microbial composition and its functions in the rumen, and subsequently influence the rumen resistome.

## Discussion

The rumen, an important organ of ruminants, is inhabited by a complex microbial system composed of various bacteria, archaea, viruses, and fungi [25]. The microbiome, which rapidly colonizes the rumen of newborn ruminants and changes with age, is critical to rumen development and ruminant growth [23, 26]. However, the rumen microbiome may also be a reservoir for antibiotic resistance and pathogenicity genes that are active after the birth of hosts [4, 27]. This study determined the temporal dynamics of the taxonomic composition of the rumen microbiome of goat kids, the results of which are in agreement with those of previous studies, revealing the colonization and development of the rumen microbiome in early life [28–31]. A strong correlation between the resistome and microbial composition in the rumen was found. Proteobacteria and Bacteroidetes members (Families: *Enterobacteriaceae*, *Pasteurellaceae*, *Prevotellaceae*, and *Bacteroidaceae*, commonly carrying ARGs) showed high proportions of the rumen microbiome of goat kids in early life. The abundance of these bacteria changed over time and was associated with dietary changes. The temporal dynamics of the rumen resistome were associated with colostrum, starter, and the maturation of the microbial community. The initial acquisition of the rumen resistome may correlate with colostrum [32], and further investigation is needed. Moreover, the priority effects and long-term persistence of the resistome need to be determined in the future.

In this study, we found that the richness of ARGs decreased and resistome structure changed with increasing age. A recent study found decreases in alpha diversity of fecal ARGs with age in dairy calves not receiving any antibiotics [32], which is consistent with our results. Correspondingly, four antibiotic compound types were observed during the milk feeding stage (d 1 to 28), while only one type was observed after d 42. Notably, the number of antibiotic resistance classes (MLS, betalactams, and Elfamycins) in the Drug types decreased with age, reflecting decreases in ARG richness. Betalactams as a major class of plasmid-mediated extended-spectrum antibiotics are used in veterinary and human medicine against bacterial infections and could improve feed utilization and performance of animals [33, 34]. However, other ARG classes, such as Aminoglycosides, Tetracyclines, and Oxazolidinone, increased over time, raising the concern that not all ARGs decrease in abundance with age.

The dynamics of age-associated ARGs were identified in our results. Before weaning when goat kids consumed milk as their primary nutrient resource, ARGs, including RPOB (daptomycin) and TUFAB (Elfamycins), GYRBA (Aminocoumarin topoisomerases), and GYRA (Fluoroquinolones), were abundant. These ARGs were also detected in cow milk samples [8], suggesting the vertical transmission of ARGs from milk to the rumen. Moreover, it is possible that these ARGs might be from an external environment. MLS23S abundance was high throughout the trial. MLS23S within the mechanism of macrolide-resistant 23S rRNA mutation could be carried in many pathogenic bacteria [35]. Other ARGs, including TETQ, TETW, TETO, TET44, TET40, and TET32 (Tetracyclines), were more abundant with age (demonstrated by both metagenomic sequencing and RT-qPCR data). The TETW gene is carried by several ruminal bacteria with an integrative and conjugative element for conjugation, recombination, and regulation, resulting in horizontal ARG transfer [36, 37]. TET32, TETW, and TETO were identified in the *Clostridium*-related human colonic anaerobe K10 and are highly distributed in the rumen of ruminants [38]. TET40 and its immediate flanking regions could disseminate genes to other bacteria in different environments [39]. Briefly, high abundances of MLS and Tetracyclines in the rumen were confirmed in other studies [7, 37]. As a result, these antibiotics widely used in food-producing animals in the US and Europe might enter and save into the rumen [40, 41].

An association between the microbiome and resistome was reported in previous studies [16, 42]. In this study, a strong correlation between the rumen resistome and microbiome was also found. The rumen microbiome in goat kids harbored 41 classes of ARGs according to the MEGARes 2.0 database when 57 classes were recruited, suggesting that the rumen microbiome carries antibiotic resistance genes. Proteobacteria, abundant in our results and a previous study [43], was identified as the most important ARG carrier, especially when goat kids consumed milk. *Enterobacteriaceae* and *Pasteurellaceae* as two main bacterial families under Proteobacteria were highly abundant on the day of kidding and strongly correlated with ARGs enriched on d1. Moreover, the major bacterial species of *Escherichia coli* and *Pseudomonas aeruginosa* were found in this study. Proteobacteria seems to be the most common feature in the gut resistome and microbiome in animals and humans [4, 8, 44], and is known for horizontally transferring DNA sequences (including ARGs) to other bacteria [45] or possible microbial diffusion between the two compartments of the digestive tract in mammalian hosts. *E. coli* was found to carry various ARGs in both the milk and rumen resistome [32, 37], suggesting that colostrum was a major source for both the rumen microbiome and resistome. *E. coli* and *P. aeruginosa* horizontally transfer ARGs to Actinobacteria, and a ‘carry-back’ mechanism for these bacteria involving conjugative transfer was reported [45]. This could explain why the abundance of *Xanthomonadaceae* (Proteobacteria) of ARGs increased with age and was associated with the ARG signatures during the starter supplementation period (d42 to d84). Overall, Proteobacteria with a strong relationship with ARGs could shape the microbial community and subsequently the antibiotic resistance structure in the early life of goat kids.

Bacteroidetes are another important group of ARG carriers [37]. The *Bacteroidaceae* family and its species, including *B. fragilis*, *B. vulgatus*, *B. heparinolyticus*, and *B. thetaiotaomicron*, were important hosts for ARGs from d1 to d14. *Bacteroidaceae* in rumen utilizes carbohydrates as an energy source [46]. The bacterial species within *Bacteroidaceae* harbor TETQ and other tetracycline resistance genes, which shows a link between the microbiome and resistome [32]. *Prevotella ruminicola* (Bacteroidetes) and *Fibrobacter succinogenes* mainly carry ARGs from d28 to d84. These two bacteria are mainly responsible for degradation and volatile fatty acid production [47, 48]. In this study, the increases in *P. ruminicola* and *F. succinogenes* were associated with starter supplementation after d 30. Moreover, the genes horizontally transferred from Proteobacteria to Bacteroidetes have been reported [49]. It is speculated that a solid diet might drive the dynamics of the bacterial community and affect horizontal ARG transfer among rumen bacteria.

The ARGs in the rumen of goat kids may be transferred from the colostrum of the mother to the rumen of goat kids. Unfortunately, we did not measure the resistome of colostrum samples in this study. However, a strong correlation of the resistome composition between paired colostrum-fecal samples was reported in calves [32], suggesting the strong possibility of transmission of ARGs from milk to the rumen. The ARGs abundant on the day of kidding were reported in raw cow’s milk [50], implying that dam’s milk may contribute to the initial acquisition of ARGs in the rumen. Moreover, Biocides, Metals, and Multi-compound types were observed in the rumen resistome before weaning, proving that these ARGs might persist in the rumen if the goat did not receive antibiotics and they might disseminate into the hindgut. Heavy metals and antibacterial biocides, which are commonly detected in animal feed and farm environments, could contribute to the promotion of ARGs in colostrum or doelings’ rumen [51, 52]. Additionally, several rumen samples were not detected in the ARGs related to metal and biocides on d 1 and 7, suggesting that individual variations have also impacts on the rumen resistome based on metagenomics. Taken together, milk, as an important nutrient source for neonatal animals, combined with environmental factors possibly served as the major source of the rumen resistome in goat kids.

Solid diet supplementation not only provides important nutrient sources for young ruminants but is also an effective feeding strategy to improve the rumen microbiome and animal performance [23, 24]. A recent study confirmed that the rumen resistome was influenced by diet through modulating the microbiome [4]. In our study, significant changes in the structure and composition of the rumen microbiome and ARGs were observed after goats consumed the starter on d 30, which is consistent with our hypothesis that diet might play a role in driving the dynamics of the rumen resistome. In addition, no antibiotics were used in animals throughout this study, indicating that ARGs could be horizontally transferred to dominant bacteria that were modulated by diet. Transfer of ARGs of Tetracycline from *Bacteroides fragilis* to *B. thetaiotaomicron* and *Prevotella ruminicola* strains through conjugative transposons [53], and genes transferred from *Bacteroides* to *Fibrobacter succinogenes* [54] were reported, which explains our results and documents that diet might be associated with ARG horizontal transfer by manipulating the microbiome. This implied that diet strategy might be an approach to manipulate rumen resistome, which should be deeply investigated in future studies. Additionally, studying the aging influences on the rumen resistome is necessary.

To further understand the mechanism of dietary factors responsible for the dynamic changes in the rumen microbiome and resistome, the functional changes in the rumen microbiome community were determined. The findings of this study revealed that the composition of enzymes associated with carbohydrates changed significantly with age. High abundances of lysozyme on the day of kidding and increased abundances of enzymes (e.g., cellulase, β-glucosidase, cellobiohydrolase, α-amylase) associated with fiber degradation during starter supplementation represent the early diet transition from colostrum to a solid diet. This phenomenon was also illustrated by the changes in the rumen microbiome with *Enterobacteriaceae*, *Pasteurellaceae,* and *Bacteroidaceae* being abundant on d1 and *Prevotellaceae* and *Fibrobacteraceae* being abundant after supplemented with a solid diet. Similarly, the ARGs carried by the microbiome showed a similar pattern. Furthermore, microbes and ARGs contained in colostrum possibly served as the initial colonization source for the rumen resistome, whereas diet and age-causing changes of certain microbial taxa might have a secondary impact on the rumen resistome.

## Conclusions

In summary, this study provides insights into the temporal dynamics of the rumen resistome in early-life goats. The richness of antibiotic-resistance genes in the rumen decreased with increasing age in the experimental antibiotic-free animals. However, some ARGs increased in abundance and expression with age. The rumen resistome was associated with the microbiota that can carry and horizontally disseminate antibiotics resistance genes. The transmission of bacteria or genes from the dam’s milk to the neonatal rumen possibly is the initial and primary source of the rumen resistome. Feeding strategies or nutritional practices in young ruminants, such as solid diet supplementation manipulating the microbiome, can influence the rumen resistome and ARG horizontal transfer. The current study complements the transmission chain of antibiotic-resistance genes in animals, which provides a clue to controlling ARGs in the gut microbiome through the feeding regime. Further research needs to determine the interactions of antibiotic resistance genes and dietary strategies to improve production performance in the livestock industry, and the potential transit of ARGs from the rumen to the hindgut in the livestock, the environment, and even humans.

## Materials and methods

### Animals and sampling

The current study was performed according to the guidance of the Animal Ethics Committee of the Chinese Academy of Agricultural Sciences (Protocol Number: AEC-CAAS-20191105; Approval date: 3 November 2019). Goat kids were raised on a goat farm (Laiwu, Shandong Province, China). All goats were reared with their dams from d 1 to d 60 and weaned and separated from their dams on d 60. A solid commercial starter was supplemented from d 31 to d 84. Accordingly, the goats that consumed dam’s milk as the only food source from d 1 to d 30, were fed both milk and starter from d 31 to d 60 and received only starter from d 61 to d 84. The detailed feeding regime is shown in Fig. 1A. The ingredients of the starter included high-quality corn, bran, soybean, DDGS, and detoxification cotton meal, with protein and NDF accounting for 18.03% and 29.62%, respectively. All goat kids in this study were healthy during our sampling period and did not receive any recorded therapeutic or prophylactic antibiotic treatments from day 1 to 84. The dams of these goat kids also did not receive any antibiotic treatments during the period of pregnancy and lactation. Regarding the management of this farm, no antibiotics were added to the diet for animals, and penicillin, streptomycin, gentamicin, and enrofloxacin were used to treat morbid animals.

Regarding the age, feeding, and diets, a total of forty-eight healthy female Laiwu black goat kids with similar body weight and feed intake at eight-time points (1, 7, 14, 28, 42, 56, 70, 84 days (d) of age) were selected. Six replicates at the designated age were slaughtered for the sampling of rumen content. Notably, on d1, goat kids suckled colostrum within half hour after born under observation and were slaughtered at the end of this day (around 20–24 h after born). Briefly, all goat kids were taken to an on-farm experimental abattoir, anesthetized using sodium pentobarbitone, and slaughtered by exsanguination from the jugular vein. After skinning and evisceration, the cardia and pylorus of the exposed rumen were sealed to reduce contamination from microbiota in other gastric parts and homogenize the rumen digesta. The rumen content samples were collected into two autoclaved 40 mL tubes (approximately 30 mL samples per tube). However, for the early age of goats (e.g., d1, d7), we tried our best to collect as much as possible. All rumen content samples were snap-frozen in liquid nitrogen and subsequently stored in a freezer at − 80 °C for further analysis.

### DNA extraction and shotgun metagenomic sequencing

Rumen content samples (*n* = 48) from goat kids at eight ages were subjected to metagenomic sequencing. To reduce the risk of environmental contamination of samples, all experimental procedures were performed in a stringent clean, and sterile class II B2 biosafety cabinet. Rumen content samples were thawed on ice, and total genomic DNA was extracted using a DNeasy PowerSoil Kit (Cat. No.12888, Qiagen, Hilden, Germany) according to the manufacturer’s instructions. Negative controls during extraction and PCR were included. DNA integrity was evaluated using 1% agarose gel electrophoresis. DNA concentration was determined using a Qubit 2.0 Fluorimeter (Invitrogen, Carlsbad, CA, USA). The DNA quality of five samples (one at d1, d7, d28, d42, and d84, respectively) was poor and subsequently removed from the library. The metagenomic library was constructed using a TruSeq DNA PCR-Free Library Preparation Kit (Illumina, San Diego, CA, USA). The quantity of the metagenomic library was evaluated with a Qubit V.2.0 Fluorimeter. Metagenomic sequencing was conducted using an Illumina HiSeq 4000 platform with 150 bp paired-end reads at the Realbio Technology Center (Shanghai, China). Sequencing data generated from shotgun metagenomes in this study have been deposited with the NCBI SRA (#PRJNA741606).

Host contaminations were removed from the raw sequencing file using KneadData by alignment to the *Capra hircus* genome. The remaining reads were then trimmed for quality using Trimmomatic (version 0.36) [55]. Next, high-quality sequencing reads were classified using Centrifuge (1.0.2-beta) by following the recommended protocol against the NCBI nt database [56].

### Resistance gene analysis

To classify the resistome of the rumen, metagenomic sequencing reads were aligned to MEGARes 2.0 (https://megares.meglab.org) by following the pipeline of AmrPlusPlus (version 2.0.2) [57]. Briefly, the host genome was removed from the raw metagenomic sequences and trimmed for quality. Then the non-host and high-quality reads were aligned to the MEGARes database 2.0 using BWA to produce SAM files for resistome analysis through ResistomeAnalyzer with default settings. The outputs of the resistome were organized to the levels of gene accession ID, gene group, mechanism of resistance, class, and antibiotic compound type. The gene accession ID level data were used to calculate the beta diversity; normalized data aggregated from the gene group-level output to the group (e.g., TETO, TETW) and class (e.g., MLS, Aminoglycosides) levels were used for heatmap or stacked bar plot visualization in this study. The sequencing reads harboring ARGs were predicted by classifying taxonomy into metagenomic assembled contigs. Briefly, the sequencing reads aligned to the database (see AmrPlusPlus 2 pipeline) were classified to the RefSeq database using Kraken2 [58, 59].

### Functional capacity analysis

To study the functional composition of rumen microbial communities, metagenomic sequence reads that had undergone the removal of the host genomes were assembled using MEGAHIT (version 1.0.6) [60] with default parameters. The assembly was predicted using Prodigal (version 2.6.3) [61], and the produced amino acid sequences were used for functional annotation. The DIAMOND program (v2.0.13) was used to map the amino acid sequences of the gene catalog into the proteins in the Kyoto Encyclopedia of Genes and Genomes (KEGG) (release 88.0) and evolutionary genealogy of genes Non-supervised Orthologous Groups (eggNOG) (v 4.5) databases with default parameters (*e* value ≤ 1e − 5) and the highest-scoring annotated hit (HSP > 60 bits) [62–64]. The KEGG database is to understand the biological system, resulting in an understanding of the functions of the microbiome in the host. The eggNOG database describes orthologous proteins and functional annotations at multiple taxonomical levels. Next, according to the above outputs, the amino acid sequences were aligned against two reference databases, the Carbohydrate-Active EnZymes database (CAZy) [65] using dbCAN2 (v2.0.11) [66] and the Clusters of Orthologous Groups of proteins database (COG) [67] using BLAST +  + (v2.11.0) [68] for functional capacity analysis. Data were normalized using the total number of trimmed reads, and normalized counts were used to calculate the Bray–Curtis dissimilarity between samples. Metagenomic-assembled contigs that were predicted to encode CAZy families were mapped to RefSeq databases using Kranken2 [58, 59]. The most abundant predicted taxon at the family level for each CAZy family was chosen for further analysis.

### Quantitative reverse transcription PCR (RT-qPCR) for ARGs

To validate metagenomic sequencing, RT-qPCR was conducted to determine the gene expression of ARGs obtained from sequencing. The ARGs, including TET44, TETQ, TETW, TETO, and TET40, were selected to represent the ARGs that gradually increased over age. Total RNA was isolated and transcribed into cDNA. The primers used for RT-qPCR are listed in Supplementary Table S4. We used 16S as a housekeeping gene and calculated 2^−ΔΔCT^ values to assess the dynamic change in the abundance of ARGs.

### Measurement of rumen enzyme activity

In addition to metagenomic sequencing, the concentrations of α-amylase and carboxymethyl cellulase (CMC) in rumen samples were measured to validate functional capacity. Rumen content samples on d1 were removed due to the insufficient sample amount. Commercial kits (Nanjing Jiancheng Bioengineering Institute, Nanjing, China) were used for measurements of the activity of α-amylase and CMC. All procedures were followed according to the manufacturer’s protocol. Briefly, the rumen fluid was centrifuged at 140 × *g* for 10 min at 4 °C, and the supernatant fluid was sonicated for 3 min and then centrifuged at 12,000 × *g* for 5 min. The measured wavelength for α-amylase and CMC was 540 nm.

### Metatranscriptomic sequencing

Metatranscriptomics is used for whole gene expression profiling and the active functions of complex microbial communities. Considering the cost of metatranscriptomics and the sample size we collected, only rumen contents on d7, d28, d56, and d84 were sequenced for the measurement of gene expression and validation for metagenomics. Total RNA was isolated from rumen samples using TRIzol reagent (Invitrogen, Carlsbad, CA, USA) according to the manufacturer’s recommendation. The integrity of the RNA was measured with an Agilent 2100 Bioanalyzer using the RNA Nano Chip (Agilent Technologies, Santa Clara, CA, USA). The quantity of RNA was determined by a Nanodrop 1000 spectrophotometer (Thermo Fisher Scientific, Waltham, MA, USA). The second type of library was constructed using rRNA depletion via the Ribo-Zero rRNA Removal Kit (H/R/M; Illumina Inc.), followed by library preparation with the TruSeq RNA Library Preparation Kit (Illumina Inc., San Diego, CA, USA) and sequencing on the Illumina NovaSeq 6000 platform (Illumina Inc.). Reads were quality-filtered and end-trimmed with Trimmomatic version 0.39. Low-quality regions (Phred quality score < Q30) were removed and reads shorter than 50 nt or duplicates were discarded. Clean reads were assembled and mapped to the MEGARes and CAZy databases as stated above.

### Statistical and bioinformatics analysis

The alpha diversity, including the Shannon index and observed bacteria or genes in each sample, was calculated to evaluate the corresponding diversities by R (v3.6.3). Principal coordinates analysis (PCoA) based on Bray–Curtis distance was performed. Then the dissimilarity of bacterial composition and ARG profiles among ages were assessed by permutational multivariate analysis of variance (PERMANOVA) using the “adonis2” function in the R “vegan” package. DESeq2 [69] was used to normalize the count data, age-dependent ARGs and CAZy families were identified by using the linear discriminant analysis (LDA) effect size (LEfSe) with default settings (e.g., LDA score > 2), and the signatures were visualized using heatmaps. To determine the relationship between rumen microbial composition and resistome, the SparCC algorithm was employed for network analysis in the R “igraph” package. Correlation coefficients below 0.6 and *p* values above 0.05 were removed. Additionally, the p values in network analysis were adjusted to avoid false positives using the FDR methods.

The correlation between the composition of the rumen resistome and that of the microbiome was determined using the Procrustes correlation [70]. The abundance matrix of ARGs and the bacterial species were Hellinger transformed, and then a PCoA for the bacterial species community and ARG abundances were performed using Bray–Curtis distances. This step was finished by using the function “vegdist” in the R “vegan” package. The function "Procrustes" in R was used for the rotation of the two dissimilarity matrixes. The correlation coefficient r of the symmetric Procrustes, the sum of squares, and the *p* value were calculated by the function “protest” in the R “vegan” package, with 9999 permutations. The outputs of Procrustes associations between the rumen microbiome and the resistome composition were visualized using the “ggplot2” package in R.

### Supplementary Information

**Additional file 1:** **Table S1.** The metagenomic sequencing statistics of raw reads. **Table S2.** Taxonomic classifications of the rumen microbiome. **Table S3.** The assembly statistics. **Table S4.** List of RT-qPCR primers. **Figure S1.** The taxonomic annotation rates of metagenomics from day 1 to 84. **Figure S2.** The abundances of major bacterial phyla in the rumen of goat kids from day 1 to 84. **Figure S3.** Alpha diversity of rumen bacteria at the family level. **Figure S4.** The major rumen archaeal phyla and genera of goat kids from day 1 to 84. **Figure S5.** The antibiotic compound types of the rumen resistome in goat kids from day 1 to 84. **Figure S6.** The alpha diversity (Shannon Index and richness) of rumen ARGs in goat kids from day 1 to 84. **Figure S7.** The gene expression of signature antibiotic resistance genes (ARGs) of the rumen in goat kids from day 1 to 84. **Figure S8.** The temporal dynamics of the bacterial phyla of ARGs in rumen resistome. **Figure S9.** The functional annotation of predicted non-redundant gene catalog at KEGG level 1. **Figure S10.** The functional annotation of predicted non-redundant gene catalog based on the functional database. (a) KEGG annotation and (b) eggNOG annotation are from metagenomic sequence; (c) KEGG annotation is from metatranscriptomics. **Figure S11.** Beta diversity of KEGG pathways of rumen microbiome in goat kids from day 1 to 84. **Figure S12.** The main CAZy classes changed with age. **Figure S13.** CAZy enzyme families in rumen metatranscriptomics. **Figure S14.** The rumen enzymes activities and the abundance of these CAZy families in metagenomics and metatranscriptomics. **Figure S15.** Network of rumen microbe-microbe interactions.

## Data Availability

Sequencing data generated from shotgun metagenomes in this study have been deposited with the NCBI SRA (#PRJNA741606) and are publicly available.

## Abbreviations

ARGs: Antibiotic resistance genes
D: Days of age
PCoA: Principal coordination analysis
PERMANOVA: Permutational multivariate analysis of variance
CAZy: Carbohydrate-active enzymes
CMC: Carboxymethyl cellulase
COG: Clusters of Orthologous Groups of proteins
GH: Glycoside hydrolase
GT: Glycosyltransferase
CBM: Carbohydrate-binding modules
PL: Polysaccharide lyases

## References

[1] Davey P, Sneddon J, Nathwani D (2010). Overview of strategies for overcoming the challenge of antimicrobial resistance. Expert Rev Clin Pharmacol.

[2] Hudson JA, Frewer LJ, Jones G, Brereton PA, Whittingham MJ, Stewart G. The agri-food chain and antimicrobial resistance: a review. Trends Food Sci Tech. 2017;69131–147. 10.1016/j.tifs.2017.09.007

[3] Godfray HC, Beddington JR, Crute IR, Haddad L, Lawrence D, Muir JF (2010). Food security: the challenge of feeding 9 billion people. Science.

[4] Auffret MD, Dewhurst RJ, Duthie CA, Rooke JA, Wallace RJ, Freeman TC, et al. The rumen microbiome as a reservoir of antimicrobial resistance and pathogenicity genes is directly affected by diet in beef cattle. Microbiome. 2017;5159. 10.1186/s40168-017-0378-z 10.1186/s40168-017-0378-z PMC5725880 29228991

[5] Pruden A, Pei R, Storteboom H, Carlson KH (2006). Antibiotic resistance genes as emerging contaminants: studies in northern Colorado. Environ Sci Technol.

[6] Felix KM, Rawlynce BC, Charles GK, Eunice M, Fidalis MD (2020). Metagenomic assessment of the rumen resistome, mobilome and stress response genes in smallholder dairy cattle in Kenya. Int J Livest Prod.

[7] Xue MY, Xie YY, Zhong YF, Liu JX, Guan LL, Sun HZ (2021). Ruminal resistome of dairy cattle is individualized and the resistotypes are associated with milking traits. Anim Microbiome.

[8] Hitch TCA, Thomas BJ, Friedersdorff JCA, Ougham H, Creevey CJ. Deep sequence analysis reveals the ovine rumen as a reservoir of antibiotic resistance genes. Environ Pollut. 2018;235571–575. 10.1016/j.envpol.2017.12.067 10.1016/j.envpol.2017.12.067 29331890

[9] Kintses B, Mehi O, Ari E, Szamel M, Gyorkei A, Jangir PK (2019). Phylogenetic barriers to horizontal transfer of antimicrobial peptide resistance genes in the human gut microbiota. Nat Microbiol.

[10] Soucy SM, Huang J, Gogarten JP (2015). Horizontal gene transfer: building the web of life. Nat Rev Genet.

[11] Zaheer R, Lakin SM, Polo RO, Cook SR, Larney FJ, Morley PS (2019). Comparative diversity of microbiomes and Resistomes in beef feedlots, downstream environments and urban sewage influent. BMC Microbiol.

[12] Boolchandani M, D'Souza AW, Dantas G (2019). Sequencing-based methods and resources to study antimicrobial resistance. Nat Rev Genet.

[13] Muurinen J, Stedtfeld R, Karkman A, Parnanen K, Tiedje J, Virta M (2017). Influence of manure application on the environmental resistome under finnish agricultural practice with restricted antibiotic use. Environ Sci Technol.

[14] Ma T, McAllister TA, Guan LL (2021). A review of the resistome within the digestive tract of livestock. J Anim Sci Biotechnol.

[15] Chambers L, Yang Y, Littier H, Ray P, Zhang T, Pruden A (2015). Metagenomic analysis of antibiotic resistance genes in dairy cow feces following therapeutic administration of third generation cephalosporin. PLoS One.

[16] Li X, Stokholm J, Brejnrod A, Alberg Vestergaard G, Russel J, Trivedi U (2021). The infant gut resistome is shaped by environmental exposures, and associates with gut bacterial maturity and asthma-associated bacterial composition. Cell Host Microbe.

[17] Vinayamohan PG, Pellissery AJ, Venkitanarayanan K. Role of horizontal gene transfer in the dissemination of antimicrobial resistance in food animal production. Curr Opin Food Sci. 2022;47100882. 10.1016/j.cofs.2022.100882

[18] Kim Y, Leung MHY, Kwok W, Fournie G, Li J, Lee PKH (2020). Antibiotic resistance gene sharing networks and the effect of dietary nutritional content on the canine and feline gut resistome. Anim Microbiome.

[19] Azad E, Derakhshani H, Forster RJ, Gruninger RJ, Acharya S, McAllister TA (2019). Characterization of the rumen and fecal microbiome in bloated and non-bloated cattle grazing alfalfa pastures and subjected to bloat prevention strategies. Sci Rep.

[20] Lourenco JM, Kieran TJ, Seidel DS, Glenn TC, Silveira MFD, Callaway TR (2020). Comparison of the ruminal and fecal microbiotas in beef calves supplemented or not with concentrate. PLoS One.

[21] Meale SJ, Li S, Azevedo P, Derakhshani H, Plaizier JC, Khafipour E, et al. Development of ruminal and fecal microbiomes are affected by weaning but not weaning strategy in dairy calves. Front Microbiol. 2016;7582. 10.3389/fmicb.2016.00582 10.3389/fmicb.2016.00582 PMC4853645 27199916

[22] Andrade BGN, Bressani FA, Cuadrat RRC, Tizioto PC, de Oliveira PSN, Mourao GB, et al. The structure of microbial populations in Nelore GIT reveals inter-dependency of methanogens in feces and rumen. J Anim Sci Biotechnol. 2020;116. 10.1186/s40104-019-0422-x 10.1186/s40104-019-0422-x PMC7038601 32123563

[23] Lv X, Chai J, Diao Q, Huang W, Zhuang Y, Zhang N (2019). The signature microbiota drive rumen function shifts in goat kids introduced to solid diet regimes. Microorganisms.

[24] Chai J, Lv X, Diao Q, Usdrowski H, Zhuang Y, Huang W (2021). Solid diet manipulates rumen epithelial microbiota and its interactions with host transcriptomic in young ruminants. Environ Microbiol.

[25] Zhao W, Abdelsattar MM, Wang X, Zhang N, Chai J (2023). In vitro modulation of rumen fermentation by microbiota from the recombination of rumen fluid and solid phases. Microbiol Spectr.

[26] Xue MY, Sun HZ, Wu XH, Liu JX, Guan LL (2020). Multi-omics reveals that the rumen microbiome and its metabolome together with the host metabolome contribute to individualized dairy cow performance. Microbiome.

[27] Donaldson SC, Straley BA, Hegde NV, Sawant AA, DebRoy C, Jayarao BM (2006). Molecular epidemiology of ceftiofur-resistant Escherichia coli isolates from dairy calves. Appl Environ Microbiol.

[28] Furman O, Shenhav L, Sasson G, Kokou F, Honig H, Jacoby S (2020). Stochasticity constrained by deterministic effects of diet and age drive rumen microbiome assembly dynamics. Nat Commun.

[29] Koringa PG, Thakkar JR, Pandit RJ, Hinsu AT, Parekh MJ, Shah RK (2019). Metagenomic characterisation of ruminal bacterial diversity in buffaloes from birth to adulthood using 16S rRNA gene amplicon sequencing. Funct Integr Genomic.

[30] Pan XY, Li ZJ, Li BB, Zhao C, Wang Y, Chen YL (2021). Dynamics of rumen gene expression, microbiome colonization, and their interplay in goats. BMC Genomics.

[31] Malmuthuge N, Liang GX, Guan LL (2019). Regulation of rumen development in neonatal ruminants through microbial metagenomes and host transcriptomes. Genome Biol.

[32] Liu JX, Taft DH, Maldonado-Gomez MX, Johnson D, Treiber ML, LemayQ DG (2019). The fecal resistome of dairy cattle is associated with diet during nursing. Nat Commun.

[33] Nobrega DB, Brocchi M (2014). An overview of extended-spectrum beta-lactamases in veterinary medicine and their public health consequences. J Infect Dev Ctries.

[34] Xu L, Zhang H, Xiong P, Zhu Q, Liao C, Jiang G. Occurrence, fate, and risk assessment of typical tetracycline antibiotics in the aquatic environment: a review. Sci Total Environ. 2021;753141975. 10.1016/j.scitotenv.2020.141975 10.1016/j.scitotenv.2020.141975 33207448

[35] Vester B, Douthwaite S (2001). Macrolide resistance conferred by base substitutions in 23S rRNA. Antimicrob Agents Chemother.

[36] Cury J, Touchon M, Rocha EPC (2017). Integrative and conjugative elements and their hosts: composition, distribution and organization. Nucleic Acids Res.

[37] Sabino YNV, Santana MF, Oyama LB, Santos FG, Moreira AJS, Huws SA (2019). Characterization of antibiotic resistance genes in the species of the rumen microbiota. Nat Commun.

[38] Melville CM, Scott KP, Mercer DK, Flint HJ (2001). Novel tetracycline resistance gene, tet(32), in the Clostridium-related human colonic anaerobe K10 and its transmission in vitro to the rumen anaerobe Butyrivibrio fibrisolvens. Antimicrob Agents Chemother.

[39] Kazimierczak KA, Scott KP, Kelly D, Aminov RI (2009). Tetracycline resistome of the organic pig gut. Appl Environ Microbiol.

[40] Food, Administration D. Antimicrobials sold or distributed for use in food-producing animals. Silver Spring : US Food and Drug Administration; 2015.

[41] Cvmp E (2011). Reflection paper on the use of macrolides, lincosamides and streptogramins (MLS) in food-producing animals in the European Union: development of resistance and impact on human and animal health. EMA.

[42] Cao J, Hu Y, Liu F, Wang Y, Bi Y, Lv N (2020). Metagenomic analysis reveals the microbiome and resistome in migratory birds. Microbiome.

[43] Stewart RD, Auffret MD, Warr A, Walker AW, Roehe R, Watson M (2019). Compendium of 4,941 rumen metagenome-assembled genomes for rumen microbiome biology and enzyme discovery. Nat Biotechnol.

[44] Hu Y, Yang X, Li J, Lv N, Liu F, Wu J (2016). The bacterial mobile resistome transfer network connecting the animal and human microbiomes. Appl Environ Microbiol.

[45] Jiang X, Ellabaan MMH, Charusanti P, Munck C, Blin K, Tong Y, et al. Dissemination of antibiotic resistance genes from antibiotic producers to pathogens. Nat Commun. 2017;815784. 10.1038/ncomms15784 10.1038/ncomms15784 PMC5467266 28589945

[46] Zhao S, Min L, Zheng N, Wang J (2019). Effect of heat stress on bacterial composition and metabolism in the rumen of lactating dairy cows. Animals (Basel).

[47] Yeoman CJ, Fields CJ, Lepercq P, Ruiz P, Forano E, White BA (2021). In Vivo Competitions between Fibrobacter succinogenes, Ruminococcus flavefaciens, and Ruminoccus albus in a gnotobiotic sheep model revealed by multi-omic analyses. MBio.

[48] Wu HM, Zhang J, Wang C, Liu Q, Guo G, Huo WJ, et al. Effects of riboflavin supplementation on performance, nutrient digestion, rumen microbiota composition and activities of Holstein bulls. Br J Nutr. 20211–8. 10.1017/S0007114520005243 10.1017/S0007114520005243 33413702

[49] Jones GH (2021). Acquisition of pcnB [poly(A) polymerase I] genes via horizontal transfer from the beta, gamma-Proteobacteria. Microb Genom.

[50] Nikoloudaki O, Lemos WJF, Campanaro S, Di Cagno R, Gobbetti M. Role prediction of Gram-negative species in the resistome of raw cow's milk. Int J Food Microbiol. 2021;340109045. 10.1016/j.ijfoodmicro.2021.109045 10.1016/j.ijfoodmicro.2021.109045 33465548

[51] Baker-Austin C, Wright MS, Stepanauskas R, McArthur JV (2006). Co-selection of antibiotic and metal resistance. Trends Microbiol.

[52] Xue HP, Wu ZW, Li LP, Li F, Wang YQ, Zhao X (2015). Coexistence of heavy metal and antibiotic resistance within a novel composite staphylococcal cassette chromosome in a staphylococcus haemolyticus isolate from bovine mastitis milk. Antimicrob Agents Ch.

[53] Scott KP (2002). The role of conjugative transposons in spreading antibiotic resistance between bacteria that inhabit the gastrointestinal tract. Cell Mol Life Sci.

[54] Montgomery L, Flesher B, Stahl D. Transfer of Bacteroides succinogenes (Hungate) to Fibrobacter gen. nov. as Fibrobacter succinogenes comb. nov. and Description of Fibrobacter intestinalis sp. nov. Int J Syst Evol. 1988;38(4):430–435. 10.1099/00207713-38-4-430

[55] Bolger AM, Lohse M, Usadel B (2014). Trimmomatic: a flexible trimmer for Illumina sequence data. Bioinformatics.

[56] Kim D, Song L, Breitwieser FP, Salzberg SL (2016). Centrifuge: rapid and sensitive classification of metagenomic sequences. Genome Res.

[57] Doster E, Lakin SM, Dean CJ, Wolfe C, Young JG, Boucher C, et al. MEGARes 2.0: a database for classification of antimicrobial drug, biocide and metal resistance determinants in metagenomic sequence data. Nucleic Acids Res. 2020;48(D1):D561-D569. 10.1093/nar/gkz1010 10.1093/nar/gkz1010 PMC7145535 31722416

[58] Wood DE, Lu J, Langmead B (2019). Improved metagenomic analysis with Kraken 2. Genome Biol.

[59] O'Leary NA, Wright MW, Brister JR, Ciufo S, McVeigh DHR, Rajput B (2016). Reference sequence (RefSeq) database at NCBI: current status, taxonomic expansion, and functional annotation. Nucleic Acids Res.

[60] Li D, Liu CM, Luo R, Sadakane K, Lam TW (2015). MEGAHIT: an ultra-fast single-node solution for large and complex metagenomics assembly via succinct de Bruijn graph. Bioinformatics.

[61] Hyatt D, Chen GL, Locascio PF, Land ML, Larimer FW, Hauser LJ. Prodigal: prokaryotic gene recognition and translation initiation site identification. BMC Bioinformatics. 2010;11119. 10.1186/1471-2105-11-119 10.1186/1471-2105-11-119 PMC2848648 20211023

[62] Buchfink B, Xie C, Huson DH (2015). Fast and sensitive protein alignment using DIAMOND. Nat Methods.

[63] Kanehisa M, Goto S, Sato Y, Kawashima M, Furumichi M, Tanabe M. Data, information, knowledge and principle: back to metabolism in KEGG. Nucleic Acids Res. 2014;42(Database issue):D199–205. 10.1093/nar/gkt1076 10.1093/nar/gkt1076 PMC3965122 24214961

[64] Huerta-Cepas J, Szklarczyk D, Forslund K, Cook H, Heller D, Walter MC, et al. eggNOG 4.5: a hierarchical orthology framework with improved functional annotations for eukaryotic, prokaryotic and viral sequences. Nucleic Acids Res. 2016;44(D1):D286–293. 10.1093/nar/gkv1248 10.1093/nar/gkv1248 PMC4702882 26582926

[65] Lombard V, Golaconda Ramulu H, Drula E, Coutinho PM, Henrissat B. The carbohydrate-active enzymes database (CAZy) in 2013. Nucleic Acids Res. 2014;42(Database issue):D490–495. 10.1093/nar/gkt1178 10.1093/nar/gkt1178 PMC3965031 24270786

[66] Zhang H, Yohe T, Huang L, Entwistle S, Wu PZ, Yang ZL (2018). dbCAN2: a meta server for automated carbohydrate-active enzyme annotation. Nucleic Acids Res.

[67] Galperin MY, Wolf YI, Makarova KS, Vera Alvarez R, Landsman D, Koonin EV (2021). COG database update: focus on microbial diversity, model organisms, and widespread pathogens. Nucleic Acids Res.

[68] Camacho C, Coulouris G, Avagyan V, Ma N, Papadopoulos J, Bealer K, et al. BLAST+: architecture and applications. BMC Bioinformatics. 2009;10421. 10.1186/1471-2105-10-421 10.1186/1471-2105-10-421 PMC2803857 20003500

[69] Love MI, Huber W, Anders S (2014). Moderated estimation of fold change and dispersion for RNA-seq data with DESeq2. Genome Biol.

[70] Gower JC. Procrustes analysis. IESBS. 2001;1812141–12143.

