# Supplementary material for: Metagenomics reveals the temporal dynamics of the rumen resistome and microbiome in goat kids
Source: Microbiome. 2024 Jan 22;12:14. doi: 10.1186/s40168-023-01733-5 (PMC10801991; doi:10.1186/s40168-023-01733-5)
Supplement: Supplementary file 2 — Additional file 1: Table S1. The metagenomic sequencing statistics of raw reads. Table S2. Taxonomic classifications of the rumen microbiome. Table S3. The assembly statistics. Table S4. List of RT-qPCR primers. Figure S1. The taxonomic annotation rates of metagenomics from day 1 to 84. Figure S2. The abundances of major bacterial phyla in the rumen of goat kids from day 1 to 84. Figure S3. Alpha diversity of rumen bacteria at the family level. Figure S4. The major rumen archaeal phyla and genera of goat kids from day 1 to 84. Figure S5. The antibiotic compound types of the rumen resistome in goat kids from day 1 to 84. Figure S6. The alpha diversity (Shannon Index and richness) of rumen ARGs in goat kids from day 1 to 84. Figure S7. The gene expression of signature antibiotic resistance genes (ARGs) of the rumen in goat kids from day 1 to 84. Figure S8. The temporal dynamics of the bacterial phyla of ARGs in rumen resistome. Figure S9. The functional annotation of predicted non-redundant gene catalog at KEGG level 1. Figure S10. The functional annotation of predicted non-redundant gene catalog based on the functional database. (a) KEGG annotation and (b) eggNOG annotation are from metagenomic sequence; (c) KEGG annotation is from metatranscriptomics. Figure S11. Beta diversity of KEGG pathways of rumen microbiome in goat kids from day 1 to 84. Figure S12. The main CAZy classes changed with age. Figure S13. CAZy enzyme families in rumen metatranscriptomics. Figure S14. The rumen enzymes activities and the abundance of these CAZy families in metagenomics and metatranscriptomics. Figure S15. Network of rumen microbe-microbe interactions. [file 40168_2023_1733_MOESM1_ESM.docx]

Table S1 The metagenomic sequencing statistics of raw reads

| Sample ID | day | % reads trimmed by Trimmomatic | % reads originating from host | Average Phred scores of clean reads |
| --- | --- | --- | --- | --- |
| LWN1 | 1 | 15.98 | 46.05 | 39.67 |
| LWN17 | 1 | 16.13 | 42.29 | 39.73 |
| LWN25 | 1 | 14.54 | 9.74 | 39.68 |
| LWN33 | 1 | 18.43 | 80.94 | 39.57 |
| LWN9 | 1 | 17.51 | 52.93 | 39.71 |
| LWN10 | 7 | 22.14 | 1.28 | 39.68 |
| LWN18 | 7 | 17.72 | 3.65 | 39.70 |
| LWN2 | 7 | 18.96 | 2.76 | 39.69 |
| LWN26 | 7 | 22.26 | 1.56 | 39.64 |
| LWN42 | 7 | 20.49 | 8.78 | 39.65 |
| LWN11 | 14 | 17.10 | 0.18 | 39.63 |
| LWN19 | 14 | 19.99 | 3.33 | 39.68 |
| LWN27 | 14 | 19.67 | 3.46 | 39.61 |
| LWN3 | 14 | 16.37 | 13.88 | 39.67 |
| LWN35 | 14 | 20.87 | 2.41 | 39.64 |
| LWN43 | 14 | 20.91 | 0.27 | 39.58 |
| LWN12 | 28 | 16.66 | 0.03 | 39.60 |
| LWN20 | 28 | 18.20 | 0.26 | 39.68 |
| LWN28 | 28 | 20.05 | 0.39 | 39.62 |
| LWN36 | 28 | 17.82 | 2.25 | 39.65 |
| LWN44 | 28 | 27.13 | 0.05 | 39.74 |
| LWN13 | 42 | 22.00 | 0.06 | 39.56 |
| LWN21 | 42 | 18.82 | 0.03 | 39.66 |
| LWN37 | 42 | 16.05 | 0.00 | 39.69 |
| LWN45 | 42 | 19.50 | 0.06 | 39.68 |
| LWN5 | 42 | 16.25 | 0.00 | 39.63 |
| LWN14 | 56 | 16.35 | 0.06 | 39.66 |
| LWN22 | 56 | 17.43 | 0.05 | 39.68 |
| LWN30 | 56 | 17.54 | 0.03 | 39.64 |
| LWN38 | 56 | 17.60 | 0.03 | 39.66 |
| LWN46 | 56 | 26.09 | 0.04 | 39.72 |
| LWN6 | 56 | 17.05 | 0.01 | 39.70 |
| LWN15 | 70 | 17.88 | 0.00 | 39.61 |
| LWN23 | 70 | 17.65 | 0.03 | 39.52 |
| LWN31 | 70 | 18.80 | 0.02 | 39.60 |
| LWN39 | 70 | 17.23 | 0.00 | 39.61 |
| LWN47 | 70 | 18.79 | 0.04 | 39.68 |
| LWN7 | 70 | 18.62 | 0.02 | 39.62 |
| LWN16 | 84 | 15.39 | 0.02 | 39.57 |
| LWN24 | 84 | 20.33 | 0.01 | 39.60 |
| LWN40 | 84 | 17.36 | 0.01 | 39.68 |
| LWN48 | 84 | 25.37 | 0.03 | 39.75 |
| LWN8 | 84 | 20.27 | 0.01 | 39.54 |

Table S2. Taxonomic classifications of the rumen microbiome

| day | Bacteria | Eukaryota | Archaea | Virus |
| --- | --- | --- | --- | --- |
| 1 | 96.6% | 1.5% | 0.2% | 1.7% |
| 7 | 97.5% | 1.3% | 0.9% | 0.3% |
| 14 | 96.7% | 1.5% | 1.4% | 0.4% |
| 28 | 95.1% | 2.6% | 1.8% | 0.5% |
| 42 | 93.6% | 4.0% | 1.7% | 0.7% |
| 56 | 93.0% | 4.6% | 1.7% | 0.7% |
| 70 | 93.4% | 4.2% | 1.7% | 0.7% |
| 84 | 91.2% | 6.1% | 1.8% | 0.9% |

Table S3 The assembly statistics

| Sample ID | day | Contigs numbers | N50 | Total size | Longest contig |
| --- | --- | --- | --- | --- | --- |
| LWN1 | 1 | 47775 | 5785 | 83377008 | 911644 |
| LWN9 | 1 | 82669 | 5913 | 134124222 | 911638 |
| LWN17 | 1 | 43513 | 16363 | 95907633 | 671087 |
| LWN25 | 1 | 43462 | 23087 | 94846151 | 671087 |
| LWN33 | 1 | 4341 | 1910 | 5057389 | 32675 |
| LWN2 | 7 | 163545 | 4793 | 240466750 | 726131 |
| LWN10 | 7 | 473109 | 1870 | 518702152 | 668409 |
| LWN18 | 7 | 195686 | 5336 | 309275322 | 1047561 |
| LWN26 | 7 | 208194 | 3282 | 277972056 | 609983 |
| LWN42 | 7 | 121824 | 7652 | 190637049 | 1285483 |
| LWN3 | 14 | 62120 | 6578 | 121123497 | 703288 |
| LWN11 | 14 | 389751 | 2508 | 480987618 | 751731 |
| LWN19 | 14 | 255337 | 3005 | 329619516 | 763848 |
| LWN27 | 14 | 356229 | 2054 | 411507672 | 313399 |
| LWN35 | 14 | 259265 | 2530 | 323973748 | 581065 |
| LWN43 | 14 | 218658 | 3268 | 301588656 | 776002 |
| LWN12 | 28 | 558175 | 1906 | 640470163 | 666713 |
| LWN20 | 28 | 541229 | 2231 | 675641963 | 552390 |
| LWN28 | 28 | 447982 | 2042 | 530973187 | 1153156 |
| LWN36 | 28 | 304787 | 2486 | 388474887 | 765979 |
| LWN44 | 28 | 484572 | 1736 | 534801384 | 727780 |
| LWN5 | 42 | 682591 | 1679 | 726398753 | 407399 |
| LWN13 | 42 | 563989 | 2037 | 663189311 | 603748 |
| LWN21 | 42 | 820774 | 1545 | 858202858 | 477863 |
| LWN37 | 42 | 197848 | 1390 | 202897133 | 396260 |
| LWN45 | 42 | 502679 | 1878 | 597014907 | 729540 |
| LWN6 | 56 | 554566 | 2119 | 687718111 | 813088 |
| LWN14 | 56 | 596732 | 1790 | 658928739 | 537150 |
| LWN22 | 56 | 583158 | 1962 | 669056499 | 599956 |
| LWN30 | 56 | 712809 | 1719 | 809038650 | 784312 |
| LWN38 | 56 | 737728 | 1544 | 782871276 | 347476 |
| LWN46 | 56 | 474426 | 2380 | 572655696 | 540096 |
| LWN7 | 70 | 514842 | 1812 | 586223871 | 638165 |
| LWN15 | 70 | 724746 | 1299 | 657981629 | 291020 |
| LWN23 | 70 | 848293 | 1456 | 860203141 | 408263 |
| LWN31 | 70 | 960919 | 1267 | 872419496 | 573665 |
| LWN39 | 70 | 568545 | 1988 | 678494232 | 702526 |
| LWN47 | 70 | 1028199 | 1325 | 974020603 | 367152 |
| LWN8 | 84 | 949474 | 1541 | 978983770 | 506278 |
| LWN16 | 84 | 866935 | 1343 | 881472149 | 583463 |
| LWN24 | 84 | 1193062 | 1065 | 986062658 | 365391 |
| LWN40 | 84 | 542996 | 1959 | 667275904 | 449880 |
| LWN48 | 84 | 248722 | 4579 | 380542073 | 777553 |

Table S4. List of RT-qPCR primers

| Gene Name | Primer ID | Primer Sequence（5‘-3’） |
| --- | --- | --- |
| TETQ | Forward | AGAATCTGCTGTTTGCCAGTG |
|  | Reverse | CGGAGTGTCAATGATATTGCA |
| TETW | Forward | GAGAGCCTGCTATATGCCAGC |
|  | Reverse | GGGCGTATCCACAATGTTAAC |
| TETO | Forward | ACGGARAGTTTATTGTATACC |
|  | Reverse | TGGCGTATCTATAATGTTGAC |
| TET44 | Forward | ATGGACTGGAGCAAGGTTTG |
|  | Reverse | TACAATTGGGGCAAGAAAGC |
| TET40 | Forward | TGCTATTGAGGAACGGCAGG |
|  | Reverse | ACAAAGCCCAGGTTGAGCTT |
| 16s | Forward | GGGTTGCGCTCGTTGC |
|  | Reverse | ATGGYTGTCGTCAGCTCGTG |

Figure S1. The taxonomic annotation rates of metagenomics from day 1 to 84


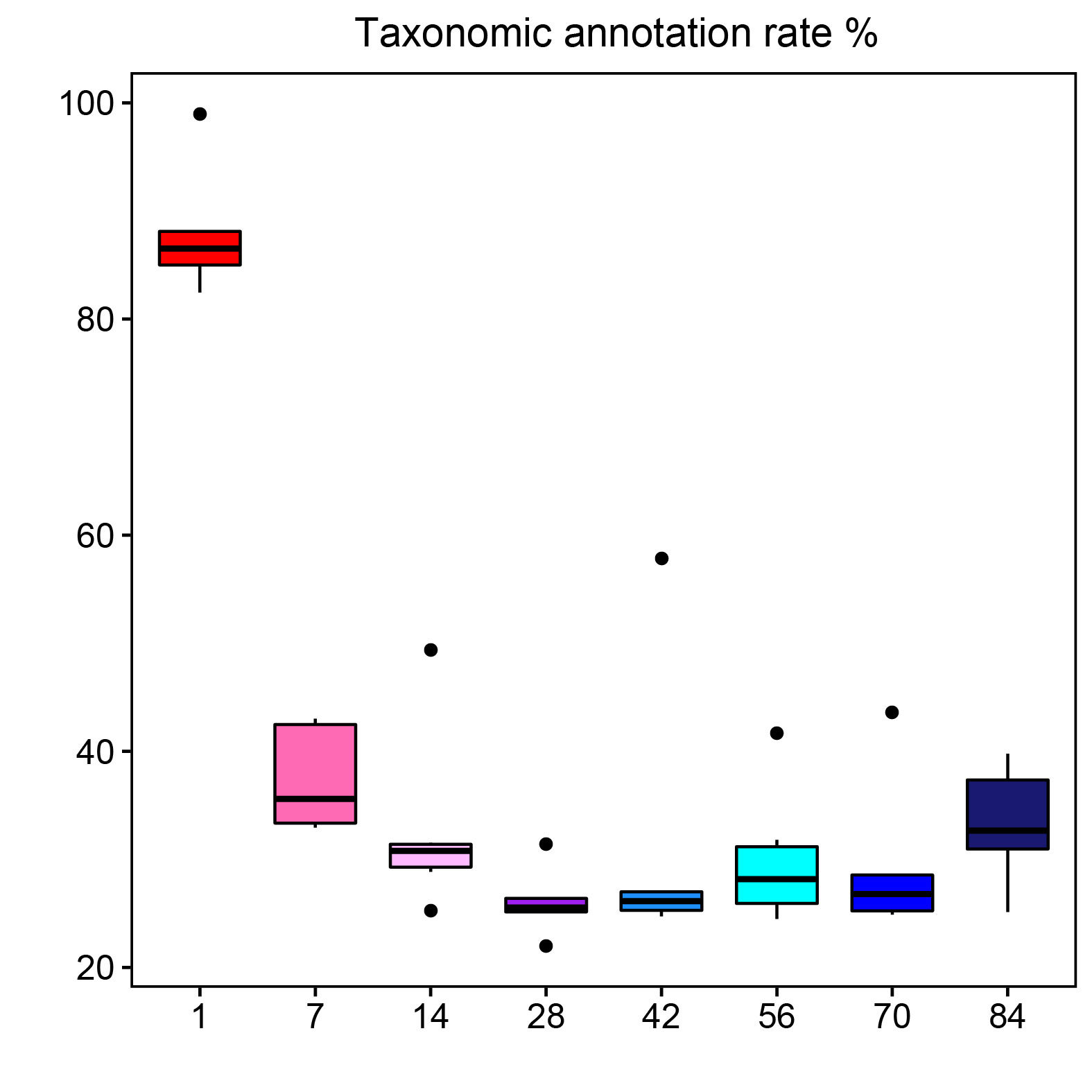


Figure S2. The abundances of major bacterial phyla in the rumen of goat kids from day 1 to 84


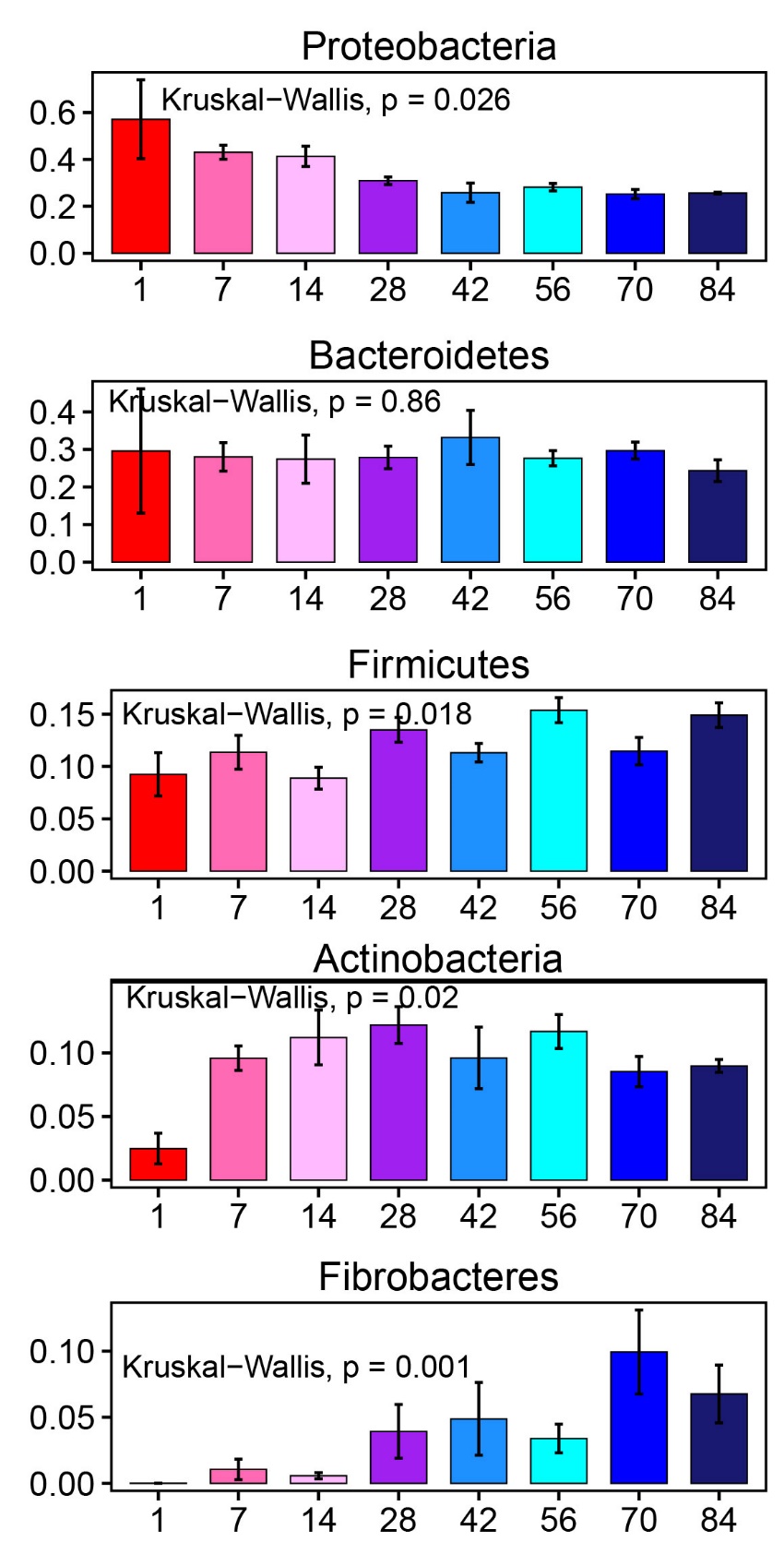


Figure S3. Alpha diversity of rumen bacteria at the family level


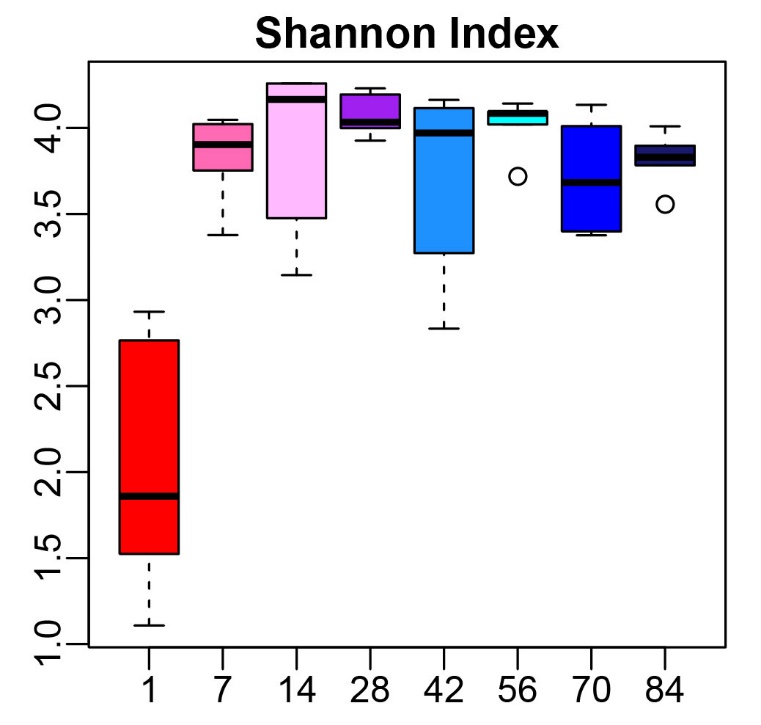


Figure S4. The major rumen archaeal phyla and genera of goat kids from day 1 to 84


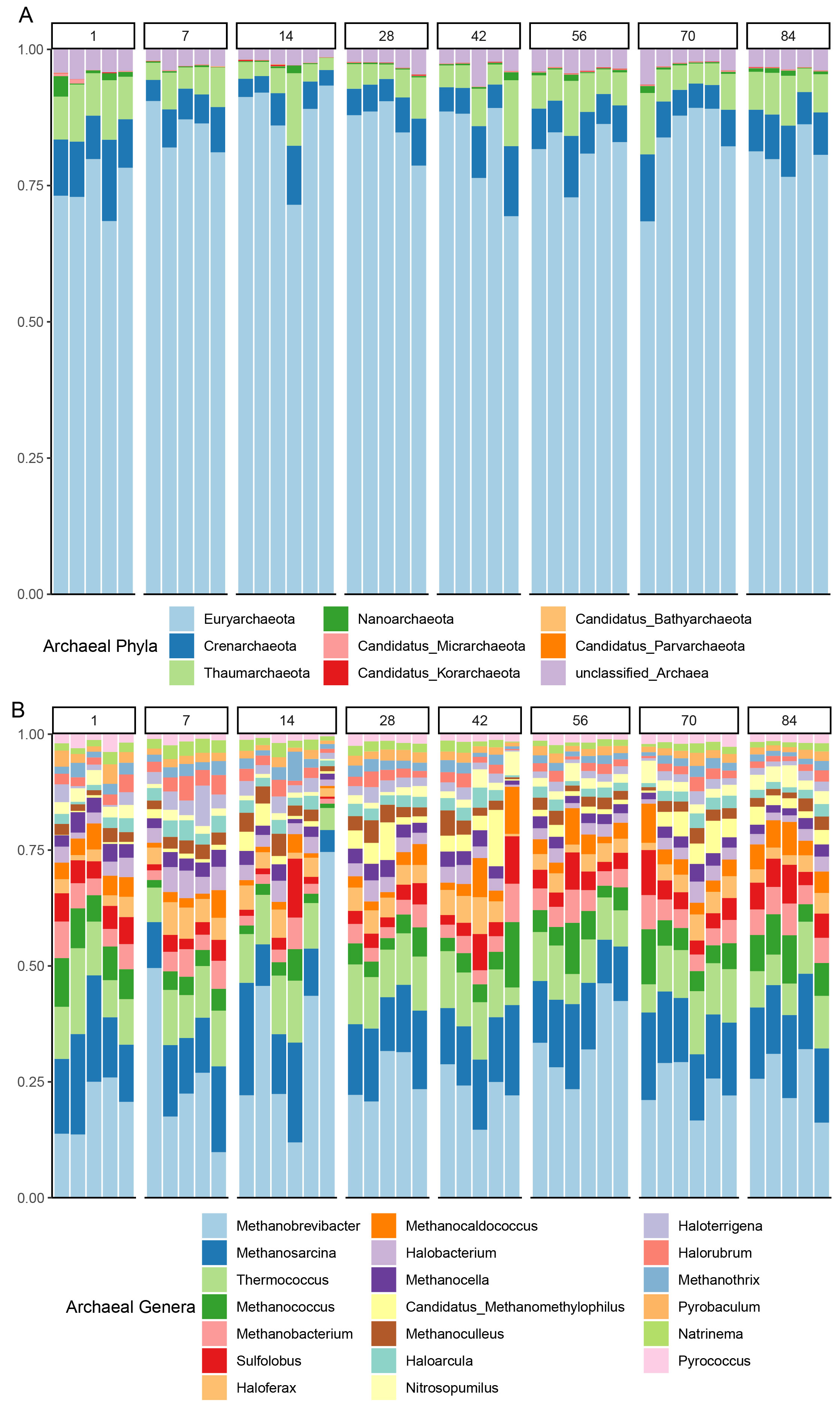


Figure S5. The antibiotic compound types of the rumen resistome in goat kids from day 1 to 84


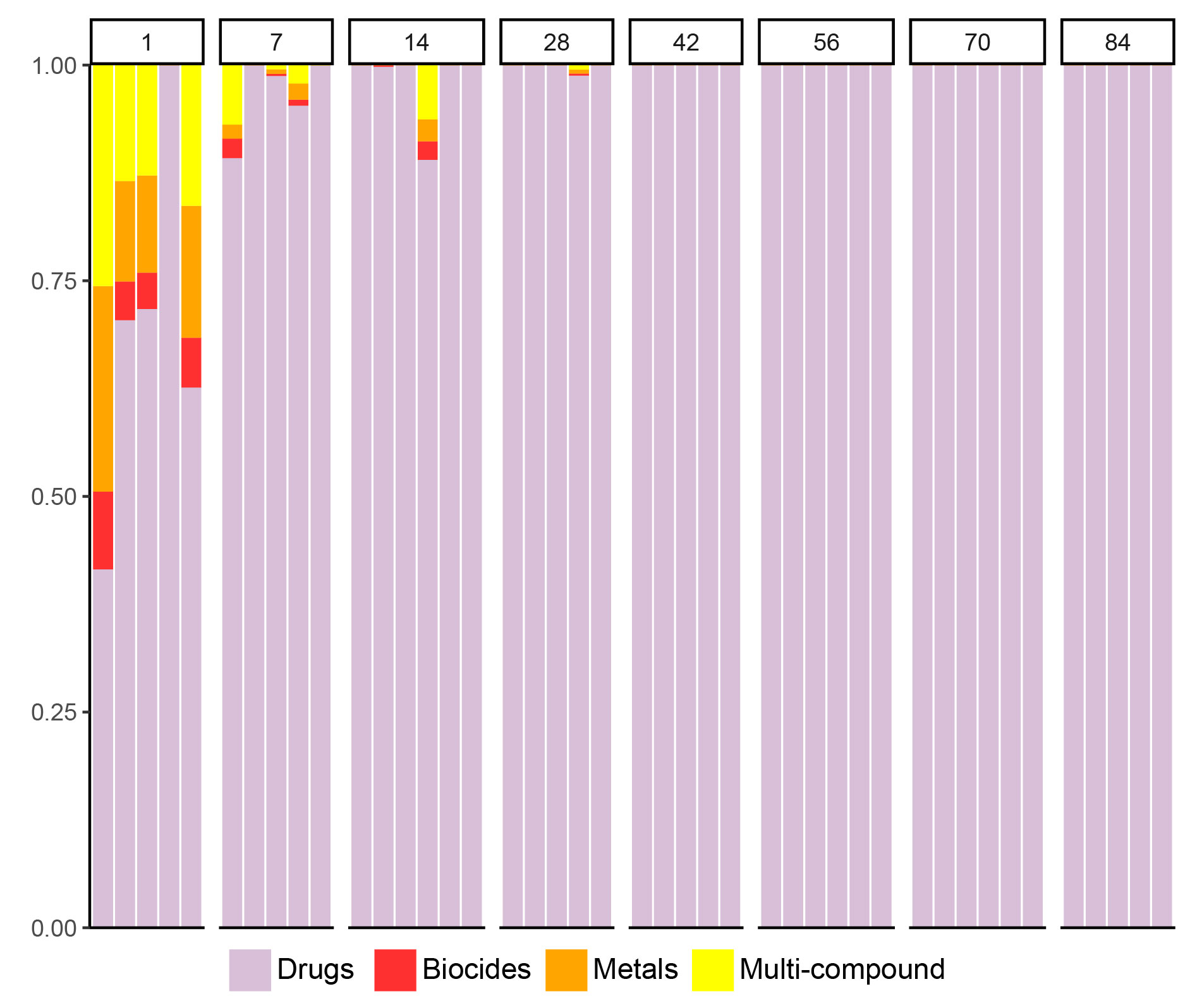


Relative abundance of antibiotic resistance genes (ARGs) at the compound type level of MEGARes 2.0. Each column represents a sample, and each bar represents an ARG compound type.

Figure S6. The alpha diversity (Shannon Index and richness) of rumen ARGs in goat kids from day 1 to 84


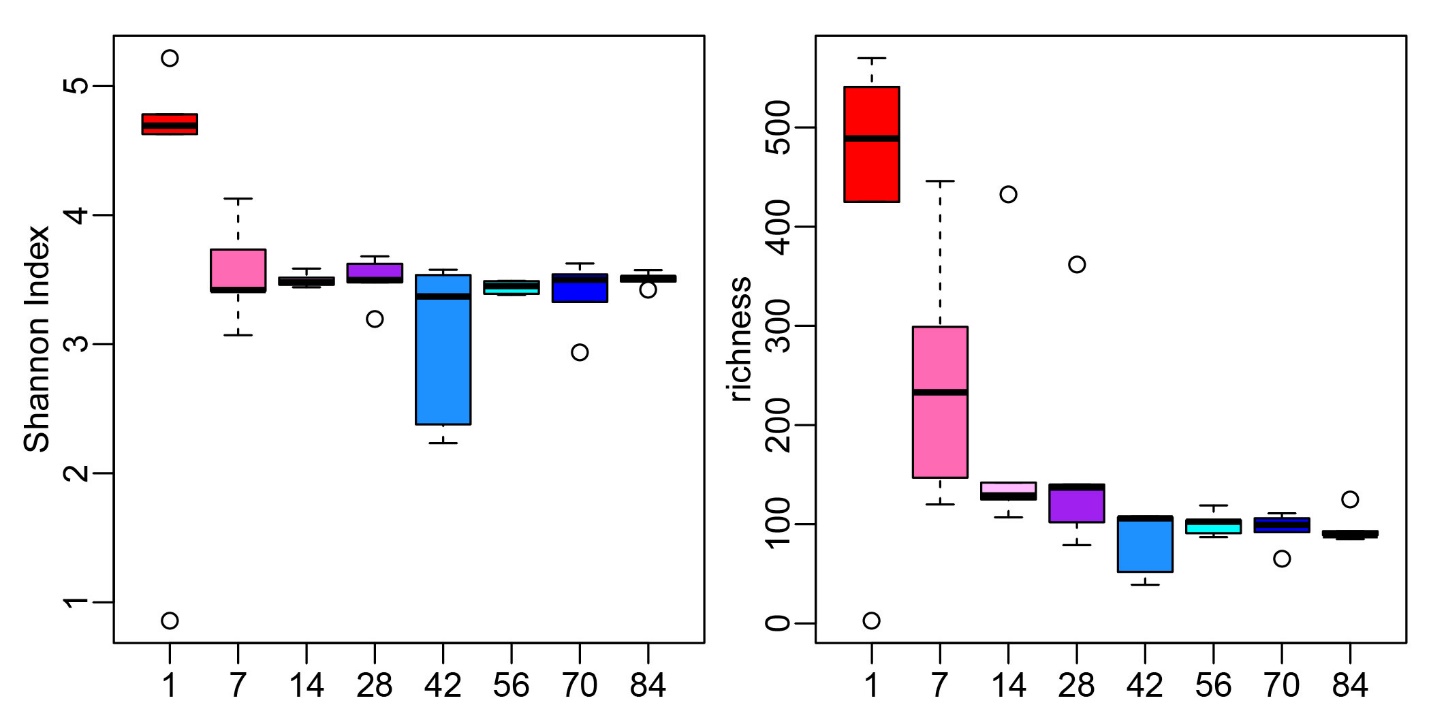


Figure S7. The gene expression of signature antibiotic resistance genes (ARGs) of the rumen in goat kids from day 1 to 84


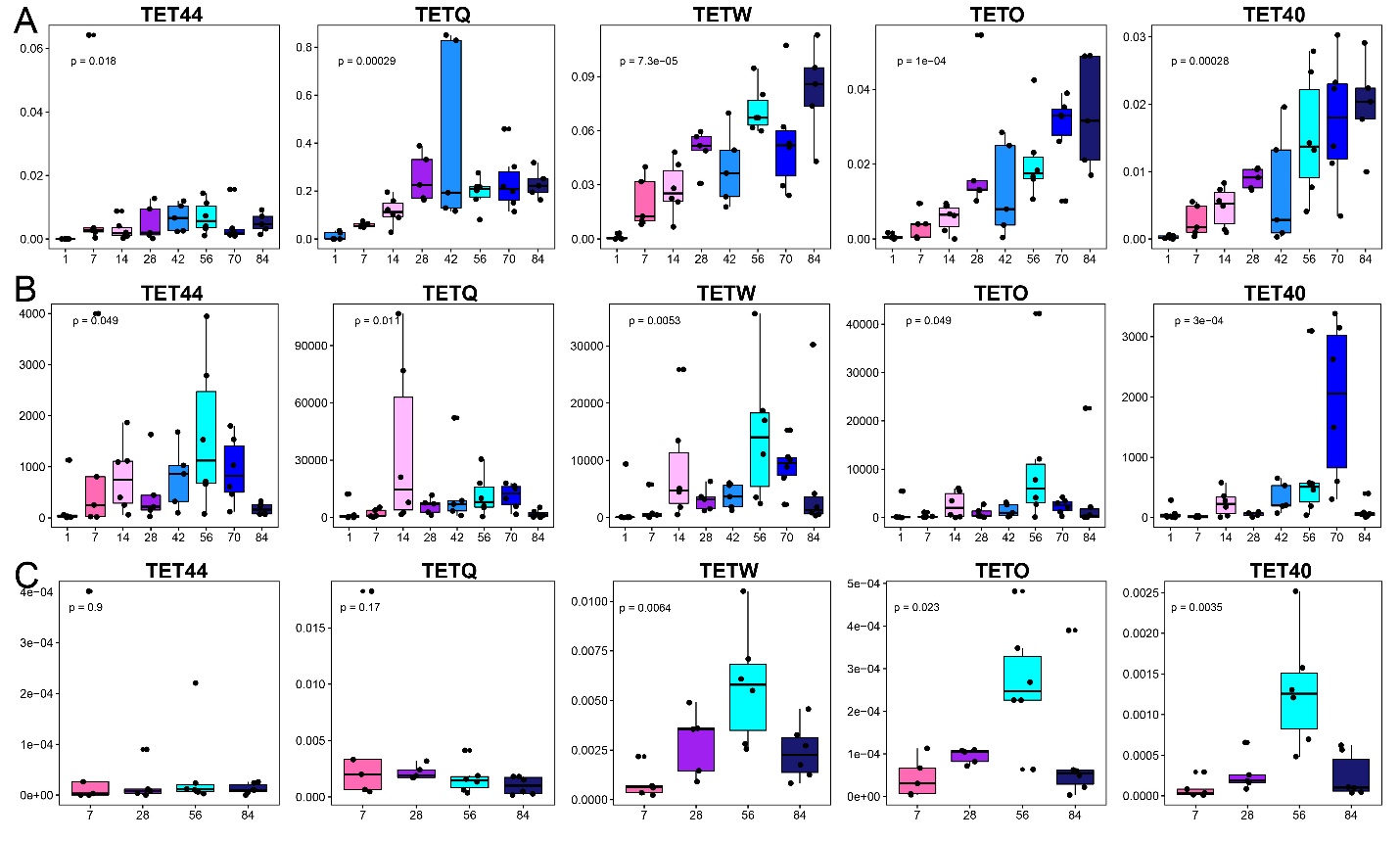


The abundances of signature ARGs from metagenomics (panel A) and metatranscriptomics (panel C) were shown, while the 2 ^-ΔΔCT^ values of ARGs from RT-qPCR were displayed on panel B.

Figure S8. The temporal dynamics of the bacterial phyla of ARGs in rumen resistome


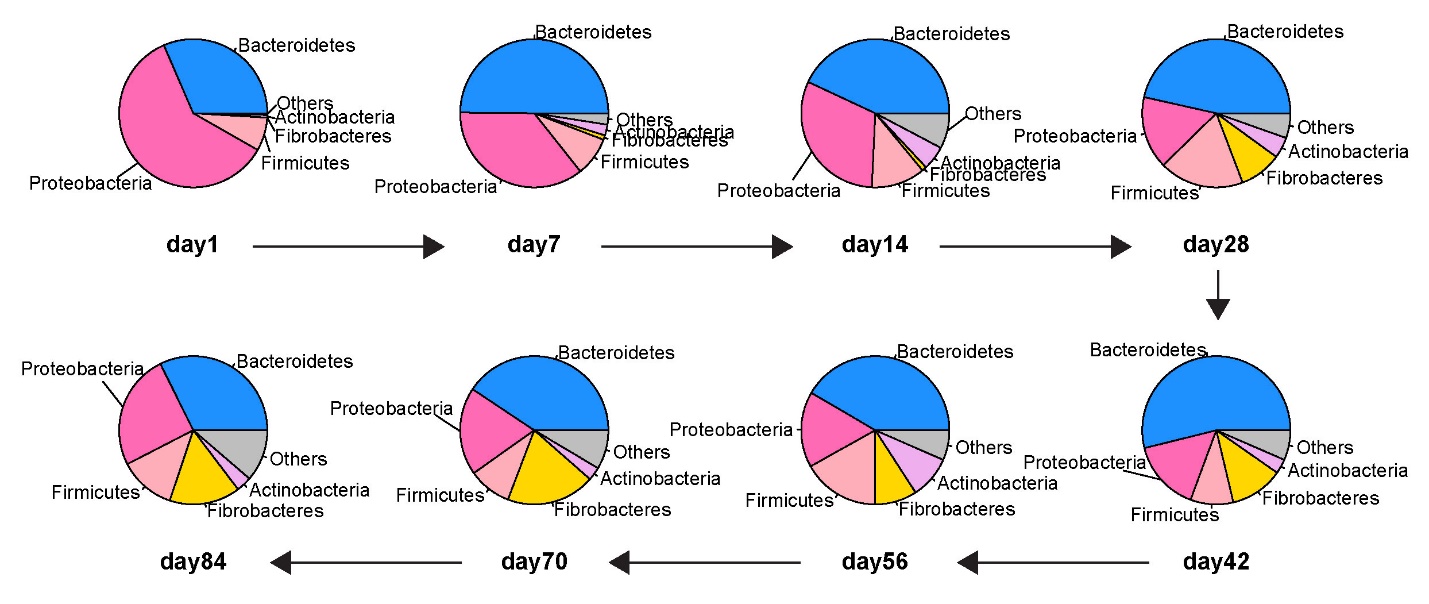


Figure S9 The functional annotation of predicted non-redundant gene catalog at KEGG level 1


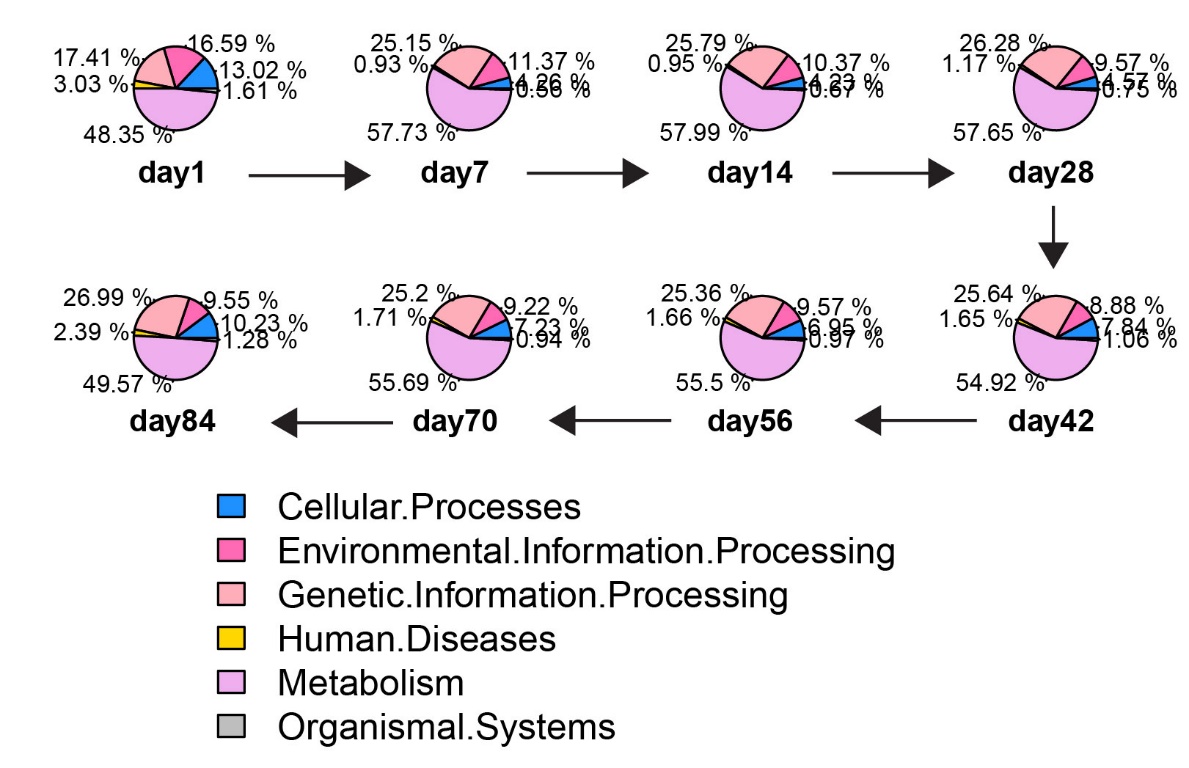


Figure S10 The functional annotation of predicted non-redundant gene catalog based on the functional database. (a) KEGG annotation and (b) eggNOG annotation are from metagenomic sequence; (c) KEGG annotation is from metatranscriptomics.


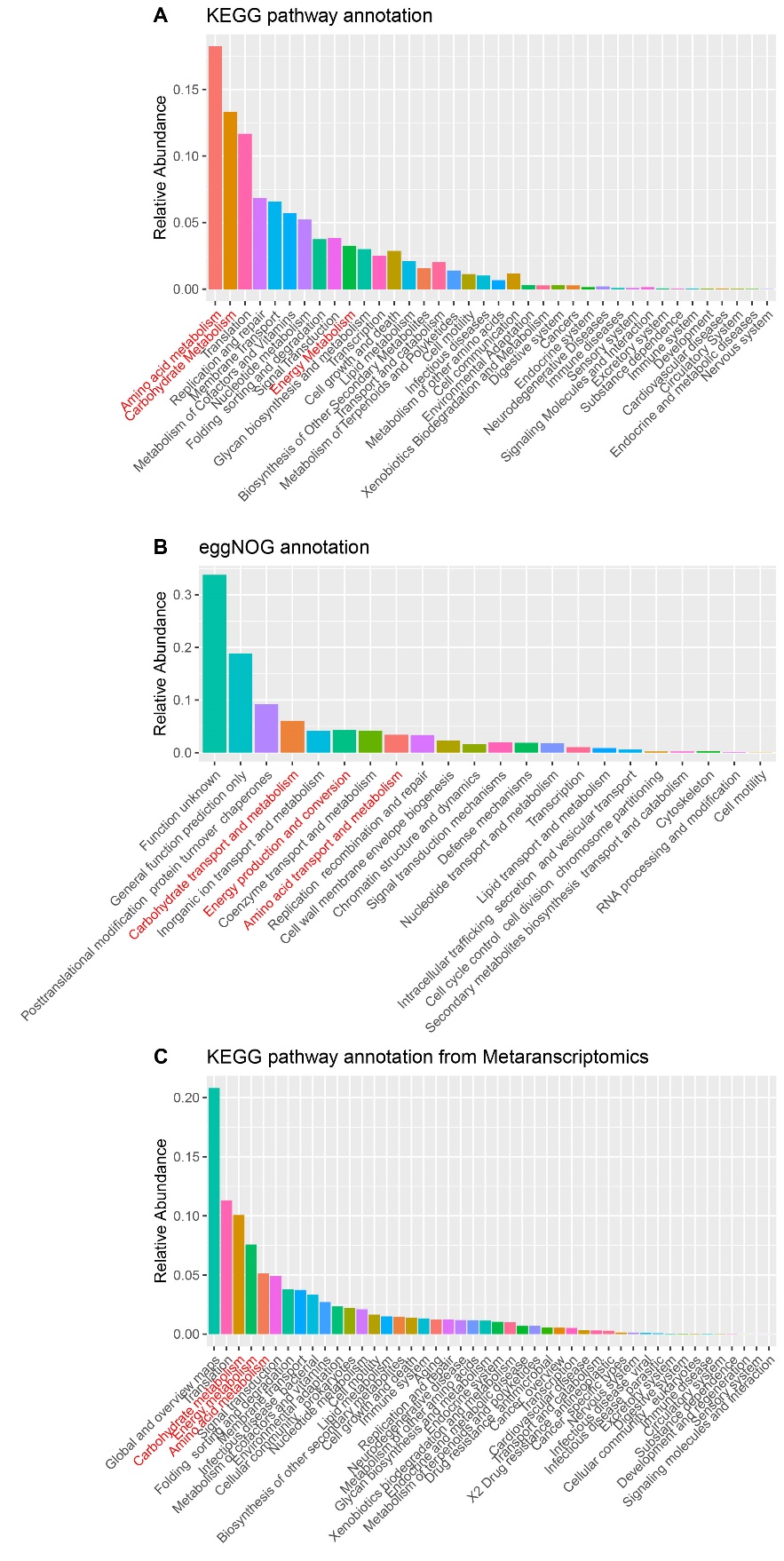


Figure S11 Beta diversity of KEGG pathways of rumen microbiome in goat kids from day 1 to 84


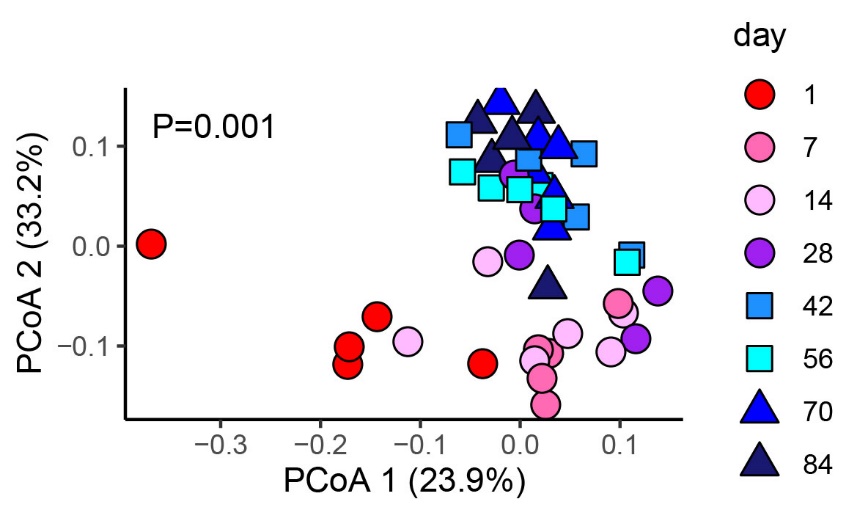


Principle Coordinate Analysis (PCoA) of Bray-Curtis distances for the KEGG pathways of rumen microbiome, showing changes over time as assessed by PERMANOVA test.

Figure S12 The main CAZy classes changed with age


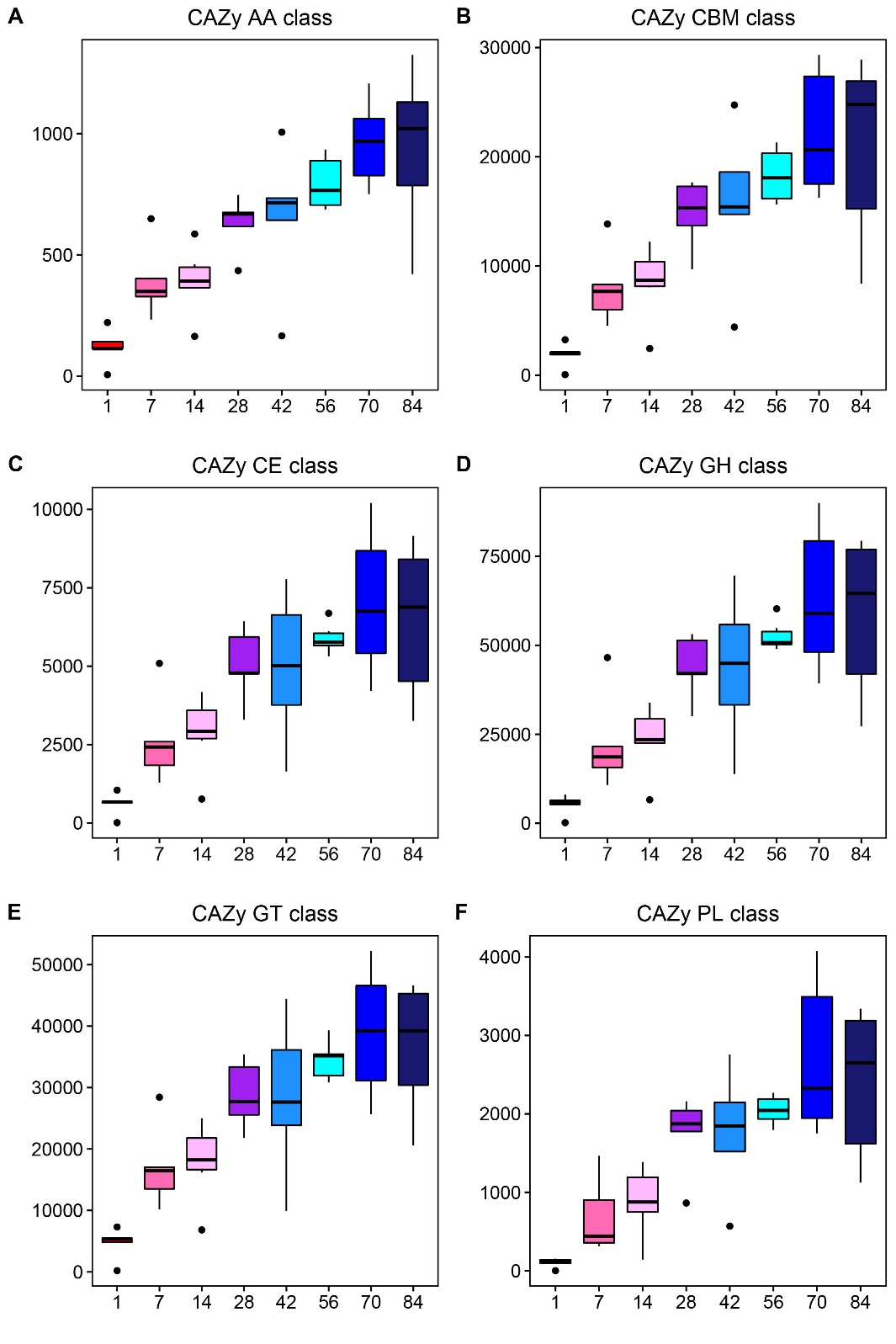


The X-axis was grouped with ages from day 1 to 84. Carbohydrate-Active EnZymes database (CAZy); Glycoside Hydrolases (GH); GlycosylTransferases (GT); Polysaccharide Lyases (PL); Carbohydrate Esterases (CE); Carbohydrate-Binding Modules (CBMs); Auxiliary Activities (AA).

Figure S13. CAZy enzyme families in rumen metatranscriptomics


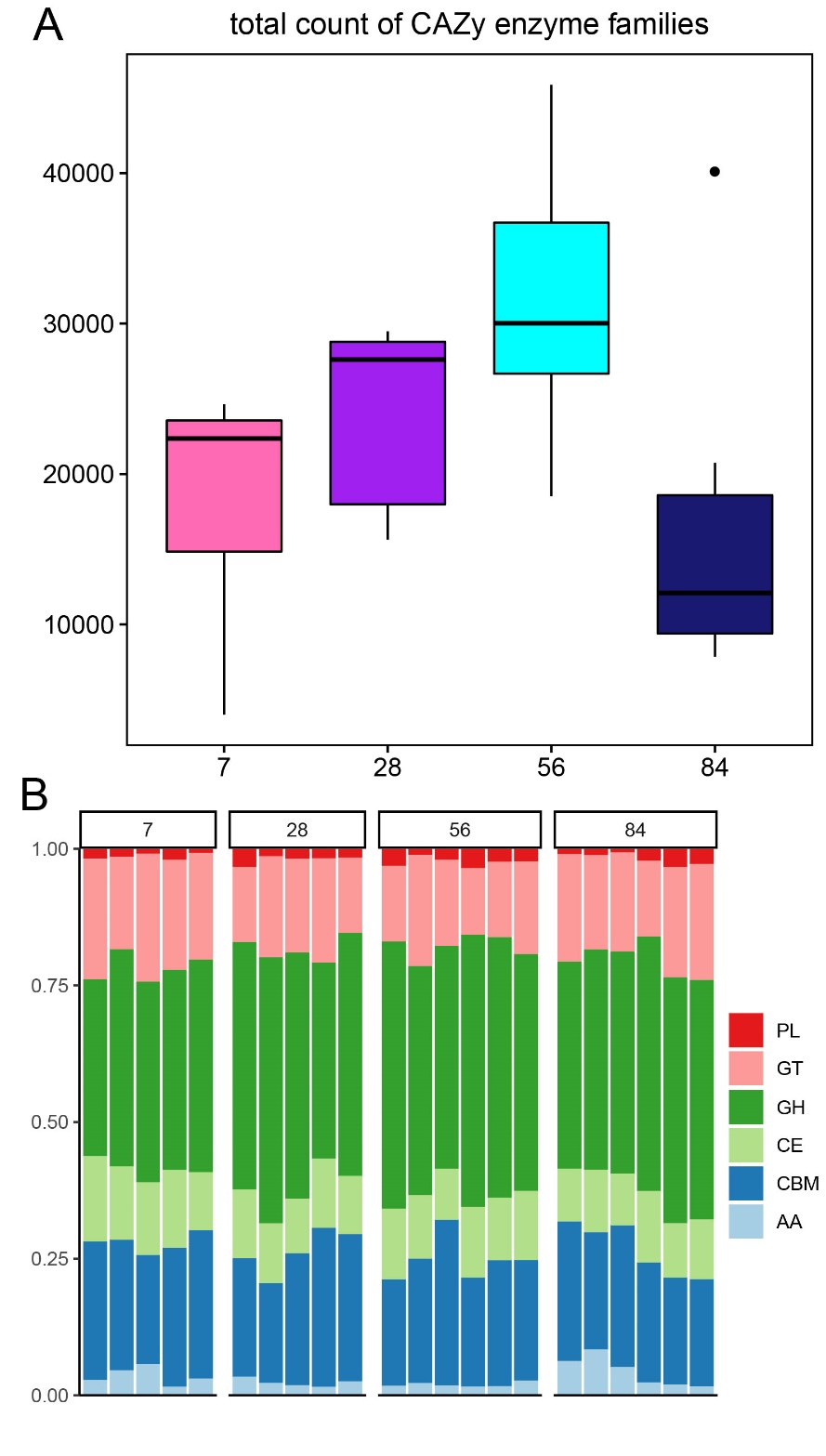


The X-axis was grouped with ages from day 7 to 84.

Carbohydrate-Active EnZymes database (CAZy); Glycoside Hydrolases (GH); GlycosylTransferases (GT); Polysaccharide Lyases (PL); Carbohydrate Esterases (CE); Carbohydrate-Binding Modules (CBMs); Auxiliary Activities (AA).

Figure S14 The rumen enzymes activities and the abundance of these CAZy families in metagenomics and metatranscriptomics


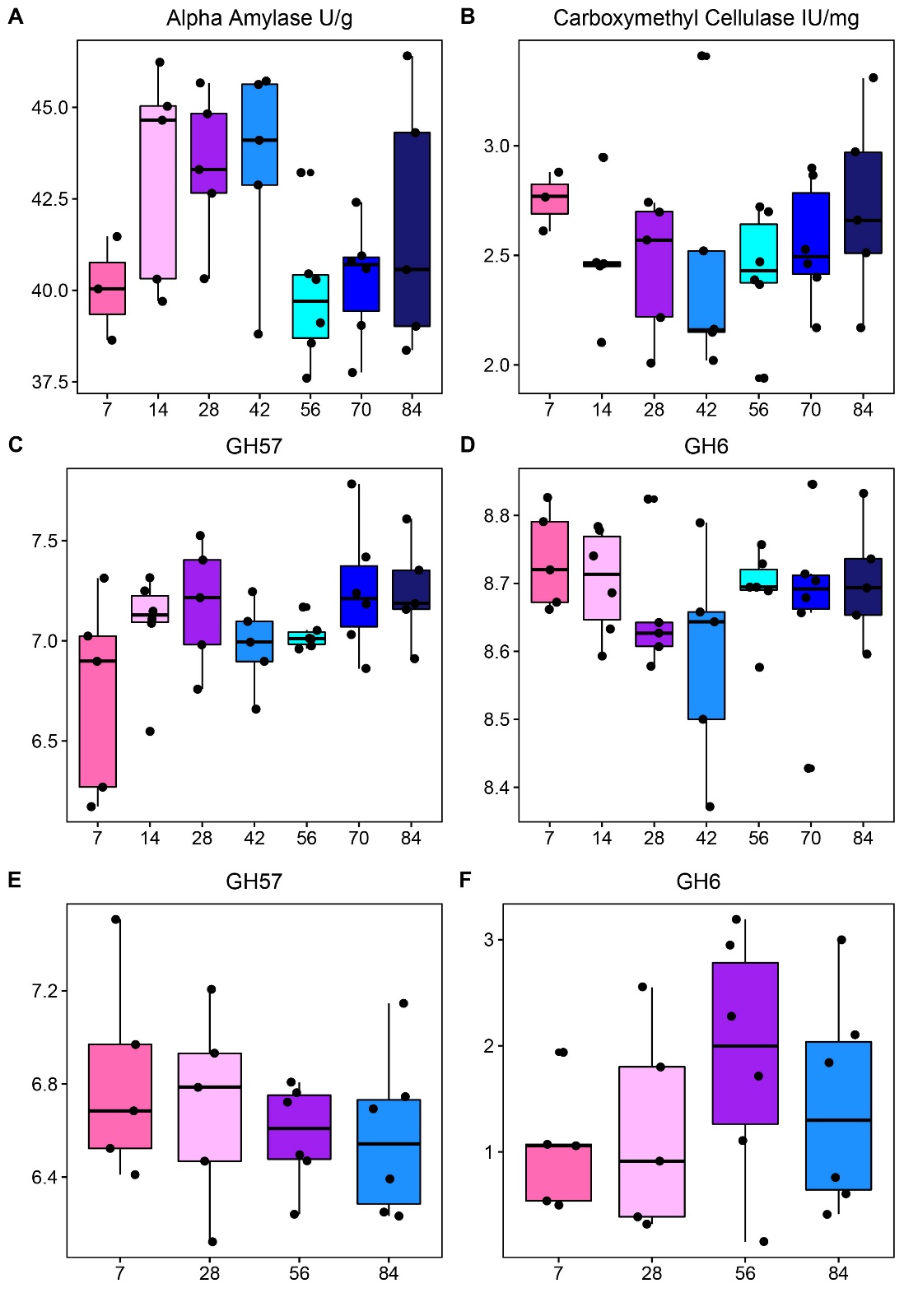


A-B: The concentration of α-amylase and carboxymethyl cellulase in rumen samples; C-D: The CAZy enzyme families in metagenomics; E-F: The CAZy enzyme families in metatranscriptomics. Carbohydrate-Active EnZymes database (CAZy);

Figure S15 Network of rumen microbe-microbe interactions


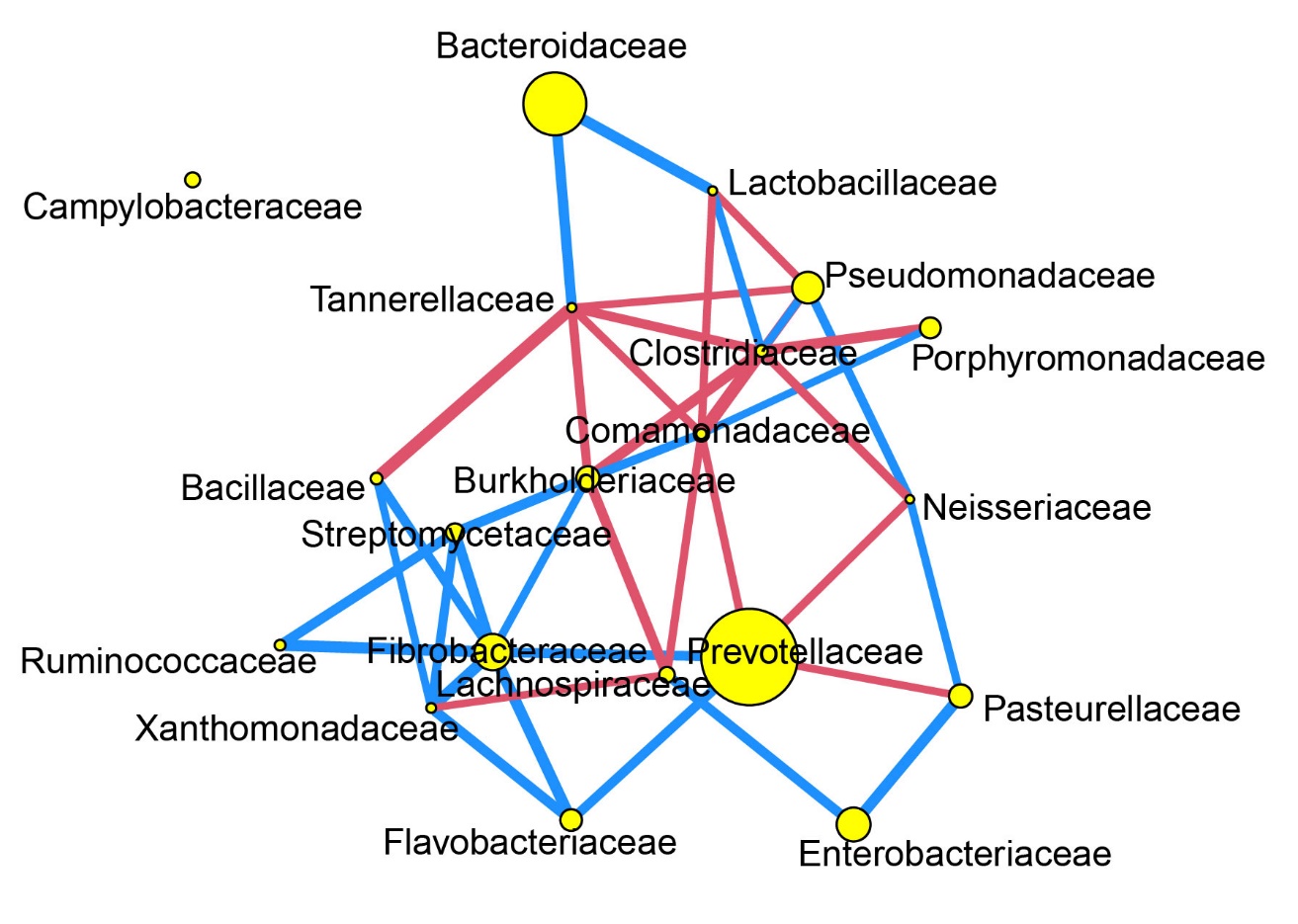


SparCC was used to calculate the relationships between bacterial taxa. The yellow circles represent the relative abundance of each bacterial family. The blue lines mean a positive correlation between bacterial families, while the red lines represent a negative correlation. The line width represents the coefficients.
